# Bright light therapy versus physical exercise to prevent co-occurring depression in adolescents and young adults with attention-deficit/hyperactivity disorder: a multicentre, three-arm, randomised controlled, pilot phase-IIa trial

**DOI:** 10.1007/s00406-024-01784-1

**Published:** 2024-04-16

**Authors:** Jutta S. Mayer, Laura Kohlhas, Jacek Stermann, Juliane Medda, Geva A. Brandt, Oliver Grimm, Adam D. Pawley, Philip Asherson, Judit Palacio Sanchez, Vanesa Richarte, Douwe Bergsma, Elena D. Koch, Adrià Muntaner-Mas, Ulrich W. Ebner-Priemer, Meinhard Kieser, Wolfgang Retz, Francisco B. Ortega, Michael Colla, Jan K. Buitelaar, Jonna Kuntsi, Josep A. Ramos-Quiroga, Andreas Reif, Christine M. Freitag

**Affiliations:** 1 Department of Child and Adolescent Psychiatry, Psychosomatics and Psychotherapy, University Hospital, Goethe University Frankfurt, Frankfurt am Main, Germany; 2 Institute of Medical Biometry, University of Heidelberg, Heidelberg, Germany; 3 Department of Psychiatry and Psychotherapy, Central Institute of Mental Health, Medical Faculty Mannheim, Heidelberg University, Mannheim, Germany; 4 Department of Psychiatry, Psychosomatic Medicine and Psychotherapy, University Hospital, Goethe University Frankfurt, Frankfurt am Main, Germany; 5 Social, Genetic and Developmental Psychiatry Centre, Institute of Psychiatry, Psychology and Neuroscience, King’s College London, London, UK; 6 Group of Psychiatry, Mental Health and Addiction, Vall d’Hebron Research Institute, Barcelona, Catalonia Spain; 7 Biomedical Network Research Centre On Mental Health (CIBERSAM), Instituto de Salud Carlos III, Madrid, Spain; 8 Department of Mental Health, Hospital Universitari Vall d’Hebron, Barcelona, Catalonia Spain; 9 Karakter Child and Adolescent Psychiatry University Centre, Nijmegen, The Netherlands; 10 Mental mHealth Lab, Institute of Sports and Sports Science, Karlsruhe Institute of Technology, Karlsruhe, Germany; 11 GICAFE “Physical Activity and Exercise Sciences Research Group”, Faculty of Education, University of Balearic Islands, Palma, Spain; 12 Department of Physical Education and Sports, Faculty of Sport Sciences, Sport and Health University Research Institute (iMUDS), University of Granada, Granada, Spain; 13 German Center for Mental Health (DZPG), partner site Mannheim, Mannheim, Germany; 14 Department of Psychiatry and Psychotherapy, University Medical Center of the Johannes Gutenberg-University, Mainz, Germany; 15 Institute for Forensic Psychology and Psychiatry, Saarland University Medical Center, Homburg/Saar, Germany; 16 CIBEROBN Physiopathology of Obesity and Nutrition, Granada, Spain; 17 Faculty of Sport and Health Sciences, University of Jyväskylä, Jyväskylä, Finland; 18 Department of Psychiatry, University Hospital Rostock, Rostock, Germany; 19 Department of Adult Psychiatry and Psychotherapy, Psychiatric University Clinic Zurich and University of Zurich, Zurich, Switzerland; 20 Department of Cognitive Neuroscience, Radboud University Medical Center, Nijmegen, The Netherlands; 21 Department of Psychiatry and Forensic Medicine, Universitat Autònoma de Barcelona, Barcelona, Catalonia Spain; 22 Fraunhofer Institute for Translational Medicine and Pharmacology ITMP, Frankfurt am Main, Germany

**Keywords:** Attention-deficit/hyperactivity disorder, Depression, Obesity, Bright light therapy, Physical exercise, m-Health, Randomised controlled trial

## Abstract

**Abstract:**

Depression is common in attention-deficit/hyperactivity disorder (ADHD), but preventive behavioural interventions are lacking. This randomised controlled, pilot phase-IIa trial aimed to study a physical exercise intervention (EI) and bright light therapy (BLT)—both implemented and monitored in an individual, naturalistic setting via a mobile health (m-health) system—for feasibility of trial design and interventions, and to estimate their effects on depressive symptoms in young people with ADHD. Two hundred seven participants aged 14–45 years were randomised to 10-week add-on intervention of either BLT (10,000 lx; daily 30-min sessions) (*n* = 70), EI (aerobic and muscle-strengthening activities 3 days/ week) (*n* = 69), or treatment-as-usual (TAU) (*n* = 68), of whom 165 (80%) were retained (BLT: *n* = 54; EI: *n* = 52; TAU: *n* = 59). Intervention adherence (i.e. ≥ 80% completed sessions) was very low for both BLT (*n* = 13, 22%) and EI (*n* = 4, 7%). Usability of the m-health system to conduct interventions was limited as indicated by objective and subjective data. Safety was high and comparable between groups. Changes in depressive symptoms (assessed via observer-blind ratings, Inventory of Depressive Symptomatology) between baseline and end of intervention were small (BLT: −0.124 [95% CI: −2.219, 1.971], EI: −2.646 [95% CI: −4.777, −0.515], TAU: −1.428 [95% CI: −3.381, 0.526]) with no group differences [*F*(2,153) = 1.45, *p* = 0.2384]. These findings suggest that the m-health approach did not achieve feasibility of EI and BLT in young people with ADHD. Prior to designing efficacy studies, strategies how to achieve high intervention adherence should be specifically investigated in this patient group.

**Trial registration:**

ClinicalTrials.gov, NCT03371810, 13 December 2017.

**Supplementary Information:**

The online version contains supplementary material available at 10.1007/s00406-024-01784-1.

## Introduction

Attention-deficit/hyperactivity disorder (ADHD) is a common neurodevelopmental condition with onset in childhood and a high rate of persistence into adulthood [1]. ADHD is associated with co-occurring mental [1] and somatic conditions [2] that add to individual disease burden [2–4]. Depression [5] and obesity [2, 6] are amongst the most common conditions, with increasing prevalence rates during transition from childhood into adulthood [2–4, 6, 7]. There is little evidence that first-line pharmacological and non-pharmacological ADHD interventions may prevent especially depression [8]. Non-adherence to medication increases during adolescence [9], further complicating effective prevention of co-occurring conditions during this particularly risky developmental phase. A wider range of non-pharmacological options that directly target known pathophysiological mechanisms of ADHD, depression and obesity is, thus, necessary.

Physical exercise attenuates the health risk of obesity [10] and is implemented in programmes to prevent and reduce obesity [11] and depression in young people [12–15]. Physical exercise is thought to directly modulate dysregulation of the dopamine system—a key pathophysiological mechanism of ADHD which also plays a role in mood disorders and obesity [16]. Following the idea of a shared disturbance of the circadian system which may link ADHD to depression [17] and obesity [18], also bright light therapy (BLT) may prevent both conditions. With morning light administration, phase delays in the sleep/wake cycle characteristic of ADHD [17] can be shifted to an earlier time. Stabilising circadian rhythms through BLT is effective in reducing depressive symptoms [19] and accumulating evidence suggests its efficacy in eating disorders and obesity [20].

To prevent increase in depressive symptoms and obesity in young people with ADHD, we developed two manualised 10-week interventions of physical exercise (EI) and BLT, both combined with a novel mobile health (m-health) system to support participants’ engagement. m-Health approaches have gained considerable popularity to promote lifestyle changes in adults [21] and adolescents [22]. There is limited evidence of their feasibility and efficacy as a tool to implement, monitor and reinforce behavioural changes in psychiatric populations, specifically young people with ADHD [23]. The m-health system included a smartphone app to deliver and prompt BLT and EI in an individual, naturalistic setting. Importantly, the app allowed online monitoring of participants’ intervention adherence, which is a critical factor for feasibility but is often not sufficiently addressed in randomised controlled trials (RCTs) [12, 24, 25]. In addition, the m-health system included a wrist-worn mobile sensor to record online physical activity and light exposure. A daily feedback mechanism provided individual reward summaries based on the data recorded online to increase engagement and motivation.

This pilot phase-IIa RCT on BLT versus EI, both in combination with m-health-based monitoring and reinforcement, addressed feasibility of trial design and interventions in adolescents and young adults with ADHD in terms of (i) recruitment and retention, (ii) data collection methods, (iii) usability of the m-health system, (iv) intervention adherence, (v) intervention integrity, and (vi) safety of interventions [26]. To inform a future definitive RCT, we established effect sizes of the primary efficacy outcome for depressive symptoms and secondary efficacy outcomes for obesity and ADHD symptoms [27].

## Methods

### Study design

This multicentre study is a pilot phase-IIa trial [27–29] with a prospective, randomised controlled, observer-blind, parallel-group design, comparing m-health based BLT and EI with treatment-as-usual (TAU) in participants with ADHD. Reporting was guided by the Consolidated Standards of Reporting Trials extension to randomised pilot and feasibility trials guidelines [26]. The study was conducted at four European centres (Goethe University Hospital Frankfurt, Germany; King’s College London, UK; Radboud University Medical Centre, Nijmegen, The Netherlands; Vall d’Hebron Research Institute, Barcelona, Spain). The study protocol [30] complied with the declaration of Helsinki (revision) and was approved by the institutional review boards of all centres [see Supplementary Information (SI) 1]. All participants provided written informed consent before their participation in the trial. For participants aged 14–17 years, written informed consent was also obtained from legal guardians.

### Participants

Eligible participants (aged 14–45 years) were recruited from clinical departments that collaborated with or were part of the participating centres, and by public announcements. All participants met DSM-5 criteria for a lifetime history of childhood-onset ADHD as well as current ADHD criteria established by psychiatric expert assessment based on structured clinical interviews. Details on procedures, inclusion, and exclusion criteria are reported in the online supporting information (SI2 and Supplementary Table 1).

### Interventions

See SI3 for detailed descriptions of the 10-week interventions and the m-health system (Supplementary Fig. 1 and 2). Briefly, BLT consisted of a daily (except Sunday) individualised, home-based 30-min exposure of white light provided by a 10,000 lx light box that supplied broadband, UV-filtered light (Philips EnergyLight HF 3419). The exact time of day of implementation (either in the morning [6–8 am] or in the evening [6–8 pm]) was determined by the chronotype of each participant.

Following the internationally accepted physical activity guidelines, EI included aerobic exercise of moderate-to-vigorous intensity and muscle-strengthening activities on 3 days a week. Based on participant’s baseline cardiorespiratory fitness, they were assigned to one of three programmes of light, moderate or high intensity. Strengthening exercises were presented in the form of video sessions on a smartphone and were executed whilst watching the videos. Aerobic activities included, for example, running, brisk walking or bicycling, and were individually chosen by participants.

Both interventions were individually incorporated into participants’ daily routines. Instruction, monitoring and feedback were realised with the m-health system comprising a smartphone (Motorola Moto G3) equipped with the m-health app (movisensXS software, movisens GmbH), and a wrist-worn light and activity sensor (LightMove 3, movisens GmbH).

TAU included stable psychopharmacotherapy for ADHD, stable medication for chronic medical conditions not interfering with interventions, individual- or group-based psychotherapy, or family support. BLT and EI were provided as add-on therapies to TAU.

### Randomisation and masking

Eligible participants were successively randomised in a 1:1:1 ratio to one of the three groups using a centralised web-based tool. Randomisation was done successively during the trial (April 2017- March 2020) in blocks with fixed length and stratified for each centre. Observers rating the severity of depressive (Inventory of Depressive Symptomatology, IDS-C_30_) and ADHD symptoms (ADHD Rating Scale) were masked to intervention allocation at all visits. Adherence to randomisation and masking procedures was monitored by an independent clinical on-site monitor throughout the trial (SI4).

### Procedures

Following screening (at visit T1) and baseline assessments (at visit T2 and with the m-health system on four days at home between T1 and T2), participants were randomised and then introduced to the respective intervention/TAU by trained study staff at T2. The devices for BLT and EI and a user’s guide were handed over. The next day, participants started either BLT or EI in addition to TAU. The control group continued with TAU. Follow-up visits occurred after 5 weeks (mid-intervention assessment, T3), after 10 weeks (end of intervention assessment, T4 including an assessment with the m-health system on four days at home), and 12–14 weeks after T4 (follow-up assessment, T5). After start of the COVID-19 pandemic, follow-up assessments were obtained via phone (SI1). Standardised motivational interviews were conducted at T2 and T3 in case of low self-reported intervention adherence (i.e. < 80% completed sessions).

### Outcome measures

#### Feasibility endpoints

Feasibility outcomes in terms of the trial design were screening, recruitment, and retention rates, duration of recruitment, and number of protocol violations. To determine the feasibility of data collection methods, number of missing data was reported for primary and secondary efficacy outcomes at each visit in the randomised population. We also reported the number of missing sensor data sets at baseline and during 10 weeks of intervention. For ratings of depressive and ADHD symptoms, we determined the percentage of observer-blind ratings at each visit and calculated interrater reliability. As a further feasibility assessment, we included participants’ adherence to interventions (BLT/ EI) based on the data recorded online with the m-health app. Specifically, we calculated the percentage of conducted sessions, the total duration of conducted sessions during 10 weeks of intervention and the mean session duration. In addition, we monitored via the sensor the mean light exposure (in lux) and physical activity (number of steps, movement acceleration) per day during 10 weeks of BLT/ EI. Participants’ adherence to interventions was also assessed retrospectively via interview at T3. Participants rated the usability of the m-health app for conducting BLT and EI using the System Usability Scale (SUS). We also monitored the wearing time of the sensor. At each visit, we recorded any change in prescribed concomitant medication and psychosocial treatment. Participants’ self-reports on continuation with BLT/ EI after T4 were included to determine acceptability of interventions. Treatment integrity was assessed via study staffs’ adherence to delivering the prescribed intervention protocol (see SI5).

All serious adverse events (SAEs) and adverse events (AEs) that caused physical or psychological harm were recorded via self-reports at each visit (SI6 and Supplementary Table 3 and 4).

#### Efficacy endpoints

The primary efficacy outcome was the change in the IDS-C_30_ total score between T2 and T4. Changes in the IDS-C_30_ total score between T2 and T5 and changes in self-reported depressive symptoms (Beck Depression Inventory, BDI) between T2 and the follow-up visits (T4, T5) were secondary outcomes. In terms of obesity, we included changes in body mass index, body fat percentage, waist circumference, and waist-to-hip ratio between baseline and follow-up visits (T4, T5). Furthermore, we assessed changes in ADHD symptoms (ADHD Rating Scale) between baseline and follow-up visits (see SI7).

### Statistical analysis

The sample size was determined to detect a clinically relevant medium effect size (*d* = 0.5) for the primary efficacy endpoint between at least one of the two interventions compared to TAU with a two-sample *t* test. As this was a pilot study, the sample size was planned at the liberal significance level of *α* = 0.10 (two-sided) and a power of 1-β = 80%, resulting in a sample size of 153 (*n* = 51 per group). A target of 219 randomisations was planned to compensate for an expected drop-out rate of 30%. For details on statistical analyses, see SI8.

#### Feasibility endpoints

We summarised feasibility outcomes using descriptive statistics including the mean (SD) for continuous data and frequencies and percentages for categorical variables. Feasibility endpoints in terms of the trial design and data collection methods were analysed in the set of all randomised participants. Participants’ adherence and acceptability of interventions, the usability and acceptability of the m-health system, and treatment integrity were evaluated in the set of participants who started the respective intervention after randomisation. Mean physical activity and light exposure were calculated for participants who wore the sensor for at least 8 h during time awake per day. Frequencies of AEs and SAEs were evaluated in the set of all randomised participants (see SI6). Additional analyses were conducted to explore predictors of participants’ adherence to interventions (BLT/ EI) by means of multivariable linear regression models. Exploratory analyses also included a cluster analysis to identify different subgroups of participants based on baseline demographic and clinical variables, which resulted in three clusters of ADHD individuals characterised by differential IQ, education of parents, age, BMI, and ADHD symptom distribution. Intervention adherence was also explored in these subgroups (for details see SI9).

#### Primary efficacy endpoint

Changes in the IDS-C_30_ between T2 and T4 were analysed as randomised in the modified intention-to-treat (mITT) population (see SI8). The mITT set consisted of all participants who were randomised, with IDS-C_30_ total scores at T2 and at least at one follow-up visit (either T3 or T4). A closed testing procedure was applied to control the overall type I error rate at 0.05. A mixed model for repeated measures (MMRM) with the restricted maximum likelihood estimation method was used to investigate the treatment effect with respect to all three groups. Within-patient errors were modelled with an unstructured covariance structure. Two group comparisons were provided by pre-defined contrasts. Baseline IDS-C_30_, age, IQ, sex, treatment, centre, visit and treatment-by-visit interaction were included as covariates. The related two-sided 95% confidence intervals for the intervention group differences were calculated. The above-described confirmatory approach controlling the type I error rate at 0.05 was pursued to enable a proof of efficacy already in this pilot study. If the effect size is *d* = 0.5 as assumed for sample size calculation, the power to reject the null hypothesis of no difference in the primary endpoint comparing one novel intervention to TAU is only 70% (instead of 80%) as planning was performed at the more liberal level of 0.10.

#### Additional analyses of the primary efficacy endpoint

Sensitivity analyses were conducted for different populations (a per-protocol population including participants without major protocol violations, complete case analysis) and applying different imputation techniques for missing values (SI8). Intervention effects on the primary efficacy endpoint were also compared between the different subgroups identified by the exploratory cluster analysis (SI9).

#### Secondary efficacy endpoints

Changes in secondary efficacy outcomes were analysed as randomised in the mITT population. The change regarding IDS-C_30_ between T2 and T5 was compared between intervention groups using the same MMRM model as in the primary analysis. This analysis was based on the follow-up data up to visit T5. Group comparisons regarding changes of all other secondary efficacy endpoints were conducted using the same MMRM models as described for the analysis of IDS-C_30_ (using the respective endpoint at baseline as a covariate instead of baseline IDS-C_30_). Baseline characteristics were summarised descriptively in the set of all randomised participants and the mITT population.

## Results

### Feasibility of trial design

**Fig. 1:**
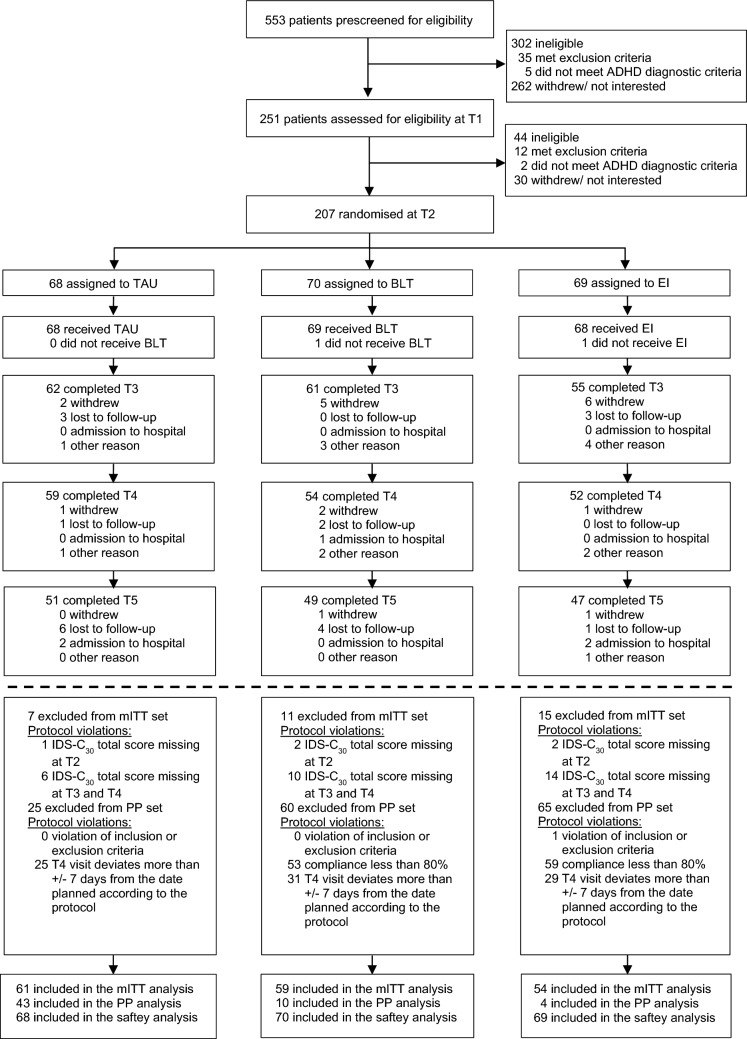
Trial profile and analysis sets. *BLT* bright light therapy, *EI* exercise intervention, *IDS-C*_*30*_ inventory of depressive symptomatology, *mITT* modified intention-to-treat set, *PP* per-protocol set, *TAU* treatment-as-usual

Between April 4, 2017 and March 31, 2020, 553 participants were screened (Supplementary Table 5), of whom 207 participants (mean age 25.8 [SD = 8.0]; range 14–44; 55.1% females [*n* = 114]) were eligible and randomly assigned to receive either BLT (*n* = 70), EI (*n* = 69), or TAU (*n* = 68) (Fig. 1). Follow-up assessments were done between May 16, 2017 and August 31, 2020. The mITT set included 174 (84%) of the randomised participants (BLT, *n* = 59, EI, *n* = 54, TAU, *n* = 61). The target sample size (*n* = 51) of the original power analysis was reached in each group for the primary efficacy analysis. Baseline characteristics are summarised in Table 1 for all randomised participants (see Supplementary Table 6 for the mITT set).

**Table 1:** Demographic and clinical characteristics at baseline of all randomised participants

	TAU (*n* = 68)	BLT (*n* = 70)	EI (*n* = 69)
Sex			
Male	36 (53%)	28 (40%)	29 (42%)
Female	32 (47%)	42 (60%)	40 (58%)
Age (years), mean (range)	25 (14–44)	27 (14–44)	26 (14–44)
IQ^a^	106 (12.7)	107 (11.9)	109 (12.7)
Highest education of patient^b^	3.6 (1.1)	3.7 (1.1)	3.6 (1.2)
Highest education of parents^c^	3.7 (0.8)	3.9 (1.1)	3.8 (1.2)
Dependent on welfare^d^	7 (10%)	7 (10%)	7 (10%)
Current school/apprenticeship/college/job attendance			
Full-time	34 (50%)	35 (51%)	39 (57%)
Part-time	14 (21%)	12 (17%)	12 (17%)
ADHD presentation			
Combined	48 (71%)	45 (65%)	45 (65%)
Predominantly inattentive	19 (28%)	23 (33%)	23 (33%)
Predominantly hyperactive-impulsive	1 (2%)	1 (1%)	1 (1%)
At least one comorbid psychiatric disorder	20 (29%)	15 (21%)	19 (28%)
Depressive disorders	13 (19%)	10 (14%)	11 (16%)
Anxiety disorders	5 (7%)	3 (4%)	2 (3%)
Obsessive–compulsive and tic disorders	2 (3%)	1 (1%)	3 (4%)
Externalising disorders	3 (4%)	0	3 (4%)
Chronic medical problems^e^	10 (15%)	20 (29%)	15 (22%)
At least one concomitant medication	52 (77%)	54 (77%)	59 (86%)
Stimulants	45 (66%)	45 (64%)	50 (73%)
Non-stimulant ADHD medication	3 (4%)	5 (7%)	4 (6%)
Anti-depressive medication	9 (13%)	15 (21%)	14 (20%)
Antipsychotic medication	2 (3%)	2 (3%)	3 (4%)
Concomitant psychosocial treatment^f^	12 (18%)	7 (10%)	13 (19%)
IDS-C_30_ total score, mean (*SD*, *n* missing data)	15.1 (11.0, 1)	14.0 (8.6, 2)	13.4 (8.4, 2)
BDI total score, mean (*SD*, *n* missing data)	12.9 (10.0, 1)	15.0 (10.7, 1)	13.0 (10.0, 4)
ADHD Rating Scale, mean (*SD*, *n* missing data)			
Total	27.1 (8.8, 1)	27.9 (9.6, 1)	25.0 (8.5, 1)
Inattentive subscale	15.3 (4.8, 1)	16.0 (5.0, 1)	14.6 (5.3, 1)
Hyperactive/ impulsive subscale	11.9 (5.3, 1)	11.9 (5.9, 1)	10.4 (5.4, 1)
BMI (kg/m^2^), mean (*SD*, *n* missing data)	24.5 (5.0, 1)	25.5 (6.0, 3)	25.8 (6.3, 1)
Obesity class I	6 (9%)	7 (10%)	5 (8%)
Obesity class II	1 (2%)	5 (7%)	5 (8%)
Obesity class III	1 (2%)	4 (6%)	0
Waist circumference (cm), mean (*SD*, *n* missing data)	81.3 (14.0, 1)	83.6 (17.2, 4)	85.7 (15.5, 1)
Waist-to-hip ratio, mean (*SD*, *n* missing data)	0.8 (0.1, 1)	0.8 (0.1, 4)	0.8 (0.1, 1)
Body fat percentage, mean (*SD*, *n* missing data)	26.0 (7.3, 0)	27.7 (7.2, 4)	27.8 (7.7, 1)
Mean number of steps per day (*SD*, *n* missing data)	15,692 (5757, 18)	14,060 (5335, 14)	15,491 (5895, 18)
Mean movement acceleration per day (*SD*,* n* missing data)	156.3 (43.9, 18)	149.1 (34.0, 14)	150.0 (37.1, 18)
Mean light exposure per day (*SD*, *n* missing data)	371.3 (468.0, 18)	398.3 (543.4, 14)	430.7 (491.3, 19)

Data are *n* (%) or mean (*SD*), unless otherwise specified

^a^Verbal and nonverbal intelligence were estimated by the vocabulary and matrix reasoning subtests of the Wechsler Adult Intelligence Scale in adults and the Intelligence Scale for Children in adolescents. The mean IQ calculated across both tasks is reported

^b^Numeric education status was calculated as follows: ISCED 0 = 0: pre-primary, ISCED 1 = 1: primary, ISCED 2A = 2: lower secondary, ISCED 3A, B, C = 3: upper secondary, ISCED 4A = 4: post-secondary, ISCED 5 A, B = 5: lower tertiary, ISCED 6 = 6: higher tertiary education

^c^Parental education status represents the mean of the biological father’s and mother’s ISCED score. If data for one biological parent were missing, the other biological parent’s score was used

^d^Dependence on welfare (yes/ no) was assessed to define socioeconomic status

^e^Chronic medical problems included, for example, asthma, hypothyroidism, allergies, headaches/ migraine, back pain, and hypertension

^f^Concomitant psychosocial treatment included psychotherapy (individual or group based), language therapy, and family-based interventions

*BDI-II* beck depression inventory, 2nd version, *BLT* bright light therapy, *BMI* body mass index, *EI* exercise intervention, *IDS-C*_*30*_ inventory of depressive symptomatology, *ISCED* international standard classification of education, *TAU* treatment-as-usual

Mean number of steps, mean movement acceleration and mean light exposure were calculated for time awake

The recruitment rate was 95% of the target sample size. Still, there were many participants who were assessed for eligibility but not enrolled (*n* = 346 of 553 screened individuals; 63%). Therefore, inclusion criteria were adapted (inclusion of participants up to 45 years old), and the study timeline was extended by 5 months. Two participants (1%) did not complete the T2 visit, and therefore did not receive their intervention after randomisation. At T3, 178 participants (total: 86%, BLT: 87%, EI: 80%, TAU: 91%) were retained, and 165 participants (total: 80%, BLT: 77%, EI: 75%, TAU: 87%) were retained at T4. Retention at follow-up (T5) was lower (total: *n* = 147, 71%, BLT: 70%, EI: 68%, TAU: 75%). Premature study termination occurred mostly because participants either declined further participation in the study (*n* = 20, 33%) or were lost to follow-up (*n* = 21, 35%). Five participants (1 adolescent, 4 adults, 8%) were admitted to psychiatric inpatient ward, and thus could not further participate. None of these admissions was due to study intervention. Protocol violations (i.e. deviation of T4 visit more than ± 7 days from the planned visit) were documented in 72% of participants (*n* = 150). These participants were excluded from the per-protocol analysis (Fig. 1).

At T4, IDS-C_30_ ratings were not available for 42 drop-outs and for additional 3 participants who did not take part in the interview (see Supplementary Table 7 and 8 for details on missing data). Across visits, the vast majority of IDS-C_30_ ratings was done observer-blind (T2: 97%; T3: 99%, T4: 96%, T5: 97%), and interrater reliability was good to excellent [*ICC*(2,1) = 0.93 (95% CI: 0.87, 0.98), see SI4 and Supplementary Table 2]. ADHD ratings were also done observer-blind (T2: 95%; T3: 97%, T4: 96%, T5: 95%) with moderate to good interrater reliability [*ICC*(2,1) = 0.80 (95% CI: 0.63, 0.93)].

### Feasibility of interventions

Feasibility of the m-health system: At baseline, sensor data were not available for 38 (18%) randomised participants. The wearing time of the sensor per day was high and comparable across groups (mean percentage, BLT, 73.9% [*SD* = 22.8]; EI, 77.5% [*SD* = 24.7]; TAU, 72.2% [*SD* = 28.7]). During intervention, sensor data sets were not available for 15 (11%) participants who received an intervention. The wearing time of the sensor dropped to 30.1% in the BLT group and to 41.4% in the EI group (Supplementary Table 9 and 10). Participants rated the m-health app as acceptable for conducting BLT (SUS, mean score 76.1), but less so for EI (mean score 65.5) (Supplementary Table 11).

### Feasibility of BLT and EI

**Fig. 2:**
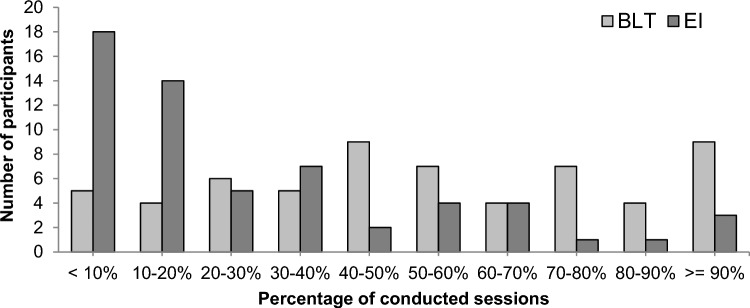
Adherence to interventions. Percentage of conducted sessions (in steps of 10%) is shown (BLT, *n* = 60; EI,* n* = 59). BLT, bright light therapy; EI, exercise intervention

In terms of intervention adherence, participants assigned to BLT completed on average 53% (*SD* = 0.4) of the prescribed 60 sessions, based on the app data (missing data sets, *n* = 9, 13%). This corresponds to a mean total duration of 960 min (*SD* = 617.5) of BLT over 10 weeks of intervention. Adherence to BLT defined as 80% or higher of completed sessions was objectively observed only for 13 participants (22%) (Fig. 2, Supplementary Table 12). In contrast, at T3, 78.9% of participants subjectively reported adherence to BLT (i.e. ≥ 80% completed sessions) over the first 5 weeks of intervention. None of the participants continued with BLT after T4 as indicated by self-reports. Mean light exposure per day was similar for both intervention groups during the intervention period (Supplementary Table 13). Exploratory regression analyses revealed that higher adherence to BLT was significantly associated with lower scores on the ADHD Rating Scale (reduced model, *estimate* = −0.9873, *t* = −2.47 *p* = 0.016). Participants’ adherence to BLT did not significantly differ between the three subgroups of participants identified by exploratory cluster analysis (SI9).

Participants assigned to EI completed on average 28% (*SD* = 28.3) of the prescribed sessions (missing data sets, *n* = 9, 13%) with only four participants (6.8%) objectively showing intervention adherence (≥80% completed sessions) (Fig. 2, Supplementary Table 14). On average, participants completed 31.2% (*SD* = 28.4) of aerobic exercise sessions [mean total duration: 354.8 min (*SD* = 331.6)] and 23.9% (*SD* = 30.3) of strengthening sessions. At T3, 50.0% of participants subjectively reported adherence to intervention over the first 5 weeks. Twenty-three participants reported that they had continued with physical exercises after T4 (mean number of sessions per month: 8.9 [*SD* = 4.2]). Descriptively, physical activity (mean number of steps and movement acceleration) was higher for participants assigned to EI than BLT during the intervention period (Supplementary Table 13). Adherence to EI was strongly associated with higher age (full model, *estimate* = 0.1587, *t* = 4.35 *p* < 0.0001), but not any other baseline demographic or clinical variables. The subgroup of participants (cluster 3) who differed predominantly in terms of age from the other two clusters showed significantly better adherence to EI compared to the younger subgroups (*p* = 0.002; see SI9).

Self-reported adherence to TAU was high (96.6%) as subjectively assessed at T3 in the control group. During study participation, changes in type of concomitant medication occurred in 39 (19%) participants who started the intervention/ TAU (BLT: *n* = 15, 22%, EI: *n* = 12, 18%, TAU: *n* = 12, 18%) whilst psychosocial treatment was stable (Supplementary Table 15).

Study staff reported that all participants were introduced to the respective intervention and the m-health system according to the pertinent standard operating procedures (SOPs). The majority of participants who reported no adherence to intervention/ TAU at T3 received motivational interviewing (BLT, 70%; EI, 78%; TAU, 25%).

Safety: AEs were reported in 99 (48%) participants with similar frequencies across groups [TAU: *n* = 29 (43%), BLT: *n* = 39 (56%), EI: *n* = 31 (45%)]. Causality with intervention was mostly considered unrelated or unlikely (*n* = 90). Intervention-related AEs (i.e. headaches) occurred in two participants from the BLT group, which led to premature study termination in one participant. SAEs were reported in six participants [TAU: *n* = 2 (2.9%), BLT: *n* = 1 (1.4%), EI: *n* = 3 (4.3%)] with no SAE deemed to be caused by study intervention (Supplementary Table 3 and 4).

### Efficacy of interventions

In terms of the primary efficacy endpoint, changes in IDS-C_30_ total score between baseline and T4 were small (see Table 2, Supplementary Fig. 3 and 4) (BLT: −0.124 [95% CI: −2.219, 1.971], EI: −2.646 [95% CI: −4.777, −0.515], TAU: −1.428 [95% CI: −3.381, 0.526]) and not different between groups [*F*(2,153) = 1.45, *p* = 0.2384]. For both interventions, intervention adherence had no effect on changes in IDS-C_30_ total score at T4. Applying last-observation-carried-forward as an imputation technique for missing values as well as complete case analysis gave very similar results. Per-protocol analyses did not show sufficient power (Supplementary Table 16 and 17). For BLT as well as EI, changes in depressive symptoms from baseline to T4 did not differ between the three clinical subgroups (SI9). With regard to secondary outcomes, there was a reduction from baseline in BMI at T4 in the BLT group whereas mean BMI increased in the TAU and EI groups. The group effect (*p* = 0.0349) was due to pairwise difference between BLT and TAU (-0.442 [95% CI: −0.776, −0.107]), and was not maintained at T5. Compared to TAU, EI had no effect on BMI at either T4 or T5. For all other secondary outcomes, no intervention effect was observed at either T4 or T5 (Table 2 and Supplementary Table 18).

**Table 2:** Mean change in primary and secondary outcomes between baseline and follow-up assessments

	BLT *n* = 59	EI *n* = 54	TAU *n* = 61
IDS-C_30_			
T4-T2	0.2 (9.1, 7)	−2.3 (6.0, 5)	−2.1 (9.8, 3)
T5-T2	1.3 (9.8, 12)	−1.9 (7.9, 9)	−0.9 (8.5, 13)
BDI-II			
T4-T2	−4.5 (9.1, 11)	−4.8 (7.4, 7)	−4.0 (10.5, 7)
T5-T2	−2.7 (9.1, 13)	−4.9 (8.4, 10)	−4.0 (9.3, 15)
BMI			
T4-T2*	−0.1 (0.9, 15)	0.2 (0.8, 10)	0.4 (0.8, 11)
T5-T2	0.3 (1.1, 21)	0.2 (0.7, 15)	0.5 (1.3, 23)
Body fat percentage			
T4-T2	0.0 (2.3, 17)	0.2 (2.2, 11)	0.3 (2.0, 11)
T5-T2	0.0 (2.7, 21)	0.6 (2.1, 15)	0.9 (2.9, 24)
Waist circumference			
T4-T2	−0.8 (4.7, 15)	−1.3 (5.0, 10)	1.2 (4.1, 11)
T5-T2	1.0 (7.7, 21)	−0.7 (7.8, 15)	1.3 (4.2, 23)
Waist-to-hip ratio			
T4-T2	0.0 (0.0, 15)	0.0 (0.1, 10)	0.0 (0.0, 11)
T5-T2	0.0 (0.1, 21)	0.0 (0.1, 15)	0.0 (0.0, 23)
ADHD rating scale			
T4-T2	−3.2 (6.3, 8)	−2.5 (8.2, 4)	−3.3 (6.7, 5)
T5-T2	−4.2 (7.4, 12)	−2.9 (8.1, 8)	−3.6 (9.1, 14)

Data are mean difference scores (T4–T2 and T5–T2) (SD, *n* missing data) for the primary and secondary outcomes analysed in the mITT set (without input values). Only participants with scores at T2 and T4 are included

*Indicates a significant group effect (*p* = 0.0349)

*BDI-II* beck depression inventory, 2nd version, *BLT* bright light therapy, *BMI* body mass index, *EI* exercise intervention, *IDS-C30* inventory of depressive symptomatology, *mITT* modified intention-to-treat, *TAU* treatment-as-usual

## Discussion

We successfully completed a European multicentre study implementing BLT and EI for prevention of depression in young people with ADHD. The large sample size allowed exploring feasibility of trial design and behavioural interventions prior to a confirmatory trial, which represents a key strength of this pragmatic pilot phase-IIa trial.

### Feasibility of trial design

Recruitment of adolescents and young adults was partly challenging, but retention rates were adequate to reach sufficient power for the primary efficacy analysis. Number of missing data was low for the primary efficacy endpoint, observer-blind assessments were done, and reliability between raters from four European sites was good to excellent. Number of missing data was also low for self-reported depressive and observed ADHD symptoms at T4. ADHD ratings were mainly done observer-blind with moderate to good interrater reliability. Body composition parameters were missing more often, and therefore findings regarding obesity need to be interpreted with more caution. Thus, the design and implementation of the trial proved overall feasible concerning recruitment rate, trial retention, masking, and data collection in terms of depression and ADHD outcome measures.

### Feasibility of interventions

With regard to interventions, the delivery of BLT and EI by trained study staff also was feasible. BLT and EI were safe with few participants reporting SAEs, which were not related to the interventions. AEs were mostly unrelated to interventions and occurred with similar rates across the three groups. Two BLT participants reported headaches, which are well-described adverse effects.

A key feature of this trial was the implementation of BLT and EI in an individual, naturalistic setting supported by an m-health system. Adequate control of adherence to behavioural interventions is challenging and often neglected in RCTs on exercise, BLT or comparable interventions in clinical populations [12, 24, 25]. This study is one of the few studies to carefully monitor and report on participants’ adherence throughout the intervention. Drop-out rates from intervention arms were comparable to those reported in EI trials for depression and other clinical populations [14] and in RCTs on psychosocial interventions in adults with ADHD [8]. Still, adherence to intervention was far lower. Based on objective m-health monitoring, only around half (BLT) to a quarter (EI) of the prescribed sessions were completed. Similarly, only 22% of BLT and 7% of EI showed intervention adherence. These findings indicate that EI and BLT as implemented in our study were not feasible in young people with ADHD.

To inform future studies, it is crucial to understand possible reasons for low intervention adherence observed in our sample of young people with ADHD, including characteristics and implementation of the interventions themselves, ADHD-related factors, and other demographic and clinical characteristics of participants. To increase motivation, we carefully matched exercise intensity to participants’ current fitness level and timing of BLT to chronotype. Still, integrating the interventions into daily routines required high commitment in terms of time and organisation. In the light of executive and reward function impairments characteristic of ADHD [16], the implementation of individualised BLT and EI in a lowly structured naturalistic setting might have been challenging. In line with this hypothesis, exploratory findings indicate that participants with more severe ADHD symptoms showed less adherence to BLT. Previous research in depressed individuals also suggests that adherence to EI is higher when conducted in inpatient settings or under supervision providing not only more structure but also social support [14]. Also, group settings have motivating effects [12, 14]. In terms of demographic characteristics, exploratory analyses identified age as a strong predictor of intervention adherence in the EI group. Better adherence to EI was also found in the subgroup of participants characterised by higher age indicating that EI as implemented in this study might be more suitable for adults rather than adolescents with ADHD. As most participants had no diagnosis of depression or obesity, participants’ motivation to engage in interventions targeting these possible future co-occurring conditions may have been limited. Importantly, a significant limitation is that the m-health system itself (especially the sensor) was not well accepted by the participants as indicated by objective and subjective data. Wearing time of the sensor was low and acceptability was limited for the more complex EI intervention that required watching videos provided by the app to learn and conduct specific exercises. This might have constrained the validity for providing daily feedback to booster motivation for a behavioural change. When designing further studies on behavioural interventions for individuals with ADHD, co-design methods including the affected individuals should be implemented to overcome the present study’s limitations regarding recruitment and acceptability [31]. Co-design may focus on intervention targets, daily routines and digital mental health technologies and should take ADHD-related factors and age of participants even more into account [32].

### Efficacy of interventions

As indicated by the main analyses and additional cluster analyses, intervention adherence had no effect on change of depressive symptoms from baseline to T4, and was overall low. Thus, the efficacy of BLT and EI to prevent depressive symptoms in young people with ADHD remains to be determined. Findings on secondary efficacy outcomes also need to be interpreted with caution given the explorative nature of the analyses. At T4, BLT showed a preventive effect on BMI compared to TAU, but this effect was small with no stability at follow-up, as BLT was not continued. Similarly, no group differences or medium pre–post effects were found for any other secondary efficacy outcome.

Future studies may overcome additional limitations of this study by the following design aspects: first, outcomes were assessed after 10 weeks of intervention and the stability of effects at 3-month follow-up. This time frame might be too short for long-term protective effects [13, 15] as well as for the development or progression of depressive symptoms given. In addition, participants were allowed to receive stable anti-depressive medication (TAU: 13%, BLT: 21%, EI: 20%) and psychosocial treatment (TAU: 18%, BLT: 10%, EI: 19%), which already may have strongly reduced their depressive symptoms, thus making it unlikely that BLT and EI might induce an even stronger reduction. Second, despite high comorbidity rates reported in the literature [3, 5, 6], in our study, participant’s depression symptoms and obesity at baseline were mostly in the normal to very mild range, possibly reflecting a recruitment bias. Importantly, the low incidence of baseline depressive symptoms might have attenuated the chance of detection of intervention effects due to a floor effect. Future studies, therefore, may focus on people with ADHD with diagnoses of depression or obesity to assess efficacy of BLT and EI in terms of treatment [33]. Third, participants showed already high physical activity before start of intervention (i.e. mean number of steps per day above the recommended criterion of 10,000) [34] and most participants had medium (*n* = 56, 28%) or high (*n* = 87, 44%) cardiorespiratory fitness. Thus, exercise-based interventions may not show an additional effect in young people with ADHD [33]. Fourth, a future efficacy trial should also measure the degree of physical activity and light exposure in the TAU group given that, in general, young people may engage in exercises and outdoor activities (see Table 1 for physical activity and light exposure assessed at baseline). The lack of controlling for these factors might have reduced the chance to find between-group differences in our pilot study.

In summary, the potential of m-health technologies to promote lifestyle changes in clinical populations has been widely discussed, but empirical evidence is scarce. We developed a novel m-health system to support BLT and EI in young people with ADHD to prevent co-occurring depression and obesity. The findings of this pilot RCT, implemented with high quality in relation to observer-blind assessments, online monitoring, and sophisticated statistical analyses, reveal that both BLT and EI conducted in an individual, naturalistic setting with an m-health system were not feasible in this group. Thus, we recommend revisions to the m-health approach and behavioural interventions for young people with ADHD, implementing co-design including affected individuals and other relevant stakeholders, and taking general (e.g. regarding the usability and acceptability of the m-health system, time commitment) as well as age-related and ADHD-specific motivational issues (e.g. by implementing more structured or group settings) even more seriously into account. This would provide an important step forward on the path toward the prevention of common co-occurring conditions in young people with ADHD.

## Conflict of interest

C.M.F. receives royalties for books on Autism Spectrum Disorder, Attention-Deficit/Hyperactivity Disorder and Major Depressive Disorder. She has served as advisor to Servier in relation to an epidemiological study in Autism Spectrum Disorder. A.R. receives honoraria for talks and/ or advisory boards from Janssen, SAGE/Biogen, Medice, Shire/Takeda, Boehringer Ingelheim, LivaNova, COMPASS, and cyclerion. J.A.R.Q. was on the speaker’s bureau and/ or acted as consultant for Janssen-Cilag, Shire, Takeda, Bial, Shionogi, Sincrolab, Novartis, BMS, Medice, Rubió, Uriach, Technofarma, and Raffo in the last 3 years. He also received travel awards (air tickets + hotel) for taking part in psychiatric meetings from Janssen-Cilag, Rubió, Shire, Takeda, Shionogi, Bial, and Medice. The Department of Psychiatry chaired by him received unrestricted educational and research support from the following companies in the last 3 years: Janssen-Cilag, Shire, Oryzon, Roche, Psious, and Rubió. J.K. has given talks at educational events sponsored by Medice; all funds are received by King’s College London and used for studies of ADHD. All other authors have no conflicts of interest to declare.

## Ethical approval

The study protocol complied with the declaration of Helsinki (revision) and was approved by the institutional review boards of all participating centres. All participants provided written informed consent before their participation in the trial. For participants aged 14–17 years, written informed consent was also obtained from legal guardians.

## Supplementary Information

Below is the link to the electronic supplementary material.

Supplementary file1 (DOCX 4236 KB)

## Data Availability

Access to a de-identified participant dataset and data dictionary is available upon reasonable request after 6 months of publication. The study protocol, statistical analysis plan, and the informed consent form can also be made available. Any such requests should be sent to the corresponding author. Requests will be assessed for scientific rigour before being granted by the trial PI. A data-sharing agreement might be required. Data will be anonymised and securely transferred.
