# Supplementary material for: Bright light therapy versus physical exercise to prevent co-occurring depression in adolescents and young adults with attention-deficit/hyperactivity disorder: a multicentre, three-arm, randomised controlled, pilot phase-IIa trial
Source: Eur Arch Psychiatry Clin Neurosci. 2024 Apr 16;275(3):653–65. doi: 10.1007/s00406-024-01784-1 (PMC11946981; doi:10.1007/s00406-024-01784-1)
Supplement: Supplementary file 1 — Supplementary file1 (DOCX 4236 KB) [file 406_2024_1784_MOESM1_ESM.docx]

**Supplementary Information**

**Title:** Bright light therapy versus physical exercise to prevent co-occurring depression in adolescents and young adults with attention-deficit/hyperactivity disorder: a multicentre, three-arm, randomised controlled, pilot phase-IIa trial

**Journal:** European Archives of Psychiatry and Clinical Neuroscience

**Authors:** Jutta S. Mayer, Laura Kohlhas, Jacek Stermann, Juliane Medda, Geva A. Brandt, Oliver Grimm, Adam D. Pawley, Philip Asherson, Judit Palacio Sanchez, Vanesa Richarte, Douwe Bergsma, Elena D. Koch, Adrià Muntaner-Mas, Ulrich W. Ebner-Priemer, Meinhard Kieser, Wolfgang Retz, Francisco B. Ortega, Michael Colla, Jan K. Buitelaar, Jonna Kuntsi, Josep A. Ramos-Quiroga, Andreas Reif, Christine M. Freitag

**Corresponding author:** Dr. Jutta S. Mayer, Department of Child and Adolescent Psychiatry, Psychosomatics and Psychotherapy, University Hospital, Goethe University Frankfurt, Deutschordenstr. 50, 60528 Frankfurt am Main, Germany, Tel: +49 – 69 – 6301 84055, Email: jutta.mayer@ukffm.de

## **SI1. Protocol changes during the study**

The study protocol [1] was ethically reviewed and approved by the institutional review boards of all participating centres (Medical Faculty, Goethe University, Frankfurt am Main, German, No. 353/16, 13 January 2017; Vall d’Hebron Research Institute, Barcelona, Spain, No. PR(AG)105/2017, 19 April 2017; King’s College London, UK, No. 17/LO/0958, 11 July 2017; Radboud University Medical Centre, Nijmegen, The Netherlands, No. 2017-3238, 5 October 2017). Protocol amendments included minor changes and were approved by the local ethics committees during the course of the study. For example, an interim report on recruitment, compliance, and safety was presented to the Data Safety and Monitoring Board (DSMB, see 4.2) at the general assembly meeting in 2018, based on data of the 36 participants randomised as of March 2018. The DSMB recommended adapting inclusion criteria to increase number of participants. The trial was continued with minor modifications (inclusion of participants up to 45 years old; Amendment No. 4, 25/04/2018; approved on 23/07/2018 by the institutional review board at Goethe University Frankfurt). Other minor amendments included changes in the information sheets and consent forms in accordance with the General Data Protection Regulation implemented by the European Union in 2018, the inclusion of an additional questionniare (i.e., System Usability Scale) [2], and the inclusion of healthy controls for additional case-control studies.

After start of the COVID-19 pandemic, follow-up assessments were done remotely. Questionnaires were sent to the patient’s homes via mail, filled in there, and sent back to the sites. The IDS-C_30_ and ADHD Rating Scale were obtained via phone (BLT, *n* = 17, EI, *n* = 18, TAU, *n* = 23). Assessment of body composition parameters were not possible. At the Frankfurt site, m-health systems for the T4 assessment were sent via mail and sent back by participants. Due to the lockdowns, this was not possible at the other sites.

**SI2. Recruitment and diagnostic procedures**

Eligible participants were recruited by flyers, public notices, and personal contact from inpatient and outpatient departments that collaborated with or were part of the participating centres, as well as by public announcements, press releases, newspaper advertisements, and internet/ social media campaigns. All participants met DSM-5 criteria for a lifetime history of childhood onset ADHD as well as current ADHD criteria established by psychiatric expert (trained clinician or clinical researcher including psychologists and psychiatrists) assessment based on structured clinical interviews. The Kiddie-Schedule for Affective Disorders and Schizophrenia - Present and Lifetime Version (K-SADS-PL) [3] was used in adolescents to assess ADHD symptoms and comorbid conditions. To obtain as much information as possible for highly valid diagnoses, both with regard to externalising and internalising conditions, the K-SADS-PL was conducted separately with the adolescent and at least one primary caregiver. For the final rating, the trained clinician/ clinical researcher took both sources of information into account. In adults, we conducted the Diagnostic Interview for ADHD in adults (DIVA 2.0) [4]. To substantiate ADHD diagnosis in adults, we also administered the Adult ADHD Self-Report Scale Symptom Checklist [5] and the Wender-Reimherr Adult ADHD Symptom Rating Scale [6]. In adults, we conducted the Structured Clinical Interview for DSM-IV Axis I and II Disorders (SCID-I and part on Borderline personality disorder of SCID-II) [7] in order to establish comorbid psychiatric diagnoses. All interviews and questionnaires were adjusted to DSM-5 criteria.

### **Supplementary Table 1.** Full list of inclusion and exclusion criteria

| Inclusion criteria | All participants must meet DSM-5 criteria for a lifetime history of childhood onset ADHD (DSM-5 314.00, 314.01) as well as current ADHD criteria established by a child psychiatrist, psychiatrist, or specialised clinical psychologist/ psychotherapist/ researcher. |
| --- | --- |
|  | Age 14 - 45 years |
|  | Written informed consent of the legal caretakers of the participant (< 18 years old) and, if possible, written assent of the participant (< 18 years old) himself |
|  | Written informed consent of the participant (>= 18 years old) |
|  | Stable TAU comprising pharmacotherapy, group based or individual cognitive behavioural therapy (not including elements of BLT or EI). |
|  | Normal or corrected to normal vision |
|  | Ability to understand, read, write, and speak fluently in the language of the study site |
|  | Ability to regularly and reliably attend appointments |
| Exclusion criteria | IQ < 75 (measured by WAIS-IV [8] or WISC-IV [9] vocabulary and matrix reasoning subtests) |
|  | Any severe comorbid psychiatric disorder implying additional psychopharmacotherapy or psychiatric intervention involving day-care/ inpatient treatment at start of study or during the study, especially a diagnosis of bipolar disorder, schizophrenia, autism spectrum disorder, schizoaffective disorder, or organic psychiatric disorder (current or lifetime) |
|  | Any severe medical or neurological condition interfering with interventions^a^ |
|  | Any severe medical or neurological condition not allowing BLT or EI^a^ |
|  | Use of antipsychotic or anti-epileptic medication, photos-sensitising medication (e.g., Lithium, St. John's Wort) |
|  | Substance use disorder (DSM-5) or dependency (DSM-5) |
|  | History of epilepsy |
|  | Acute suicidal ideation |
|  | Pregnancy |
|  | Participant related to sponsor, primary investigator, or study staff |
|  | Participation in other clinical trials and observation period of competing trials (participation in other studies is permitted if the respective study is no medication or psychotherapy trial, and if its aims do not interfere with the aims of the present study) |
|  | No participant is allowed to enrolling in this trial more than once |

^a^With regard to comorbid medical and neurological conditions, it was essential that participants had no severe condition interfering with or not allowing either BLT (e.g. diagnosed eye condition or other diseases with effects on the retina such as Diabetes mellitus, or recent eye surgery) or EI (e.g. heart disease, high blood pressure, injuries).

## **SI3. Description of interventions**

### **3.1 Exercise intervention (EI)**

In developing the EI manual and learning videos, we followed the internationally accepted physical activity guidelines ([http://www.health.gov/paguidelines/](http://www.health.gov/paguidelines)). These guidelines are based on a systematic review of > 2000 references and are the basic platform for designing any exercise intervention. In accordance with the guidelines, most of the time during the exercise sessions was spent in aerobic exercise of moderate-to-vigorous intensity and strength activities. The EI consisted in training three days a week for ten weeks. Participants performed three days of proposed aerobic activities and on two of these days they also did muscle-strengthening exercises. Specifically, a training day consisted of: (1) a 5-min warm-up period; (2) 10–35 min of muscle-strength training on two of the three days; (3) 20–40 min of aerobic training; and (4) 5 min of flexibility/ stretching cool-down. During the course of the ten weeks, the duration and intensity of the exercises increases gradually by combining number of exercises, repetitions, rests, and frequency.

(1) The warm-up consisted of doing an activity at a slower speed or lower intensity. A warm-up before moderate or vigorous intensity aerobic activity allows a gradual increase in heart rate and breathing at the start of the episode of activity. Warm-up included light walking and joint mobilisation of the upper (neck, shoulders) and lower limbs (hips, knees, and ankles). (2) The muscle-strength training included whole-body exercises, which incorporated push-ups, front plank, lunge, chair squat, hip thrust, etc. (3) Aerobic activities were physical activities in which people move their large muscles in a rhythmic manner for a sustained period. Aerobic activity makes a person’s heart beat more rapidly to meet the demands of the body’s movement. Running, brisk walking, bicycling, playing basketball, dancing, and swimming are all examples of aerobic activities. (4) A cool-down after activity allows a gradual decrease at the end of the episode. The cool-down period included breathing, stretching, and relaxing exercises.

The participants decided which days of the week they wanted to perform the physical exercises, which was entered into a weekly plan. Participants were recommended to complete training sessions separated by 48-h rest periods whenever possible. Not all participants had the same fitness level at the beginning of the intervention. Consequently, and in order to ensure adherence by all participants, we prescribed three physical exercise programmes of differing intensity based on the participant’s baseline cardiorespiratory fitness. In particular, we used the Chester Step Test to assess the baseline cardiorespiratory fitness level. The Chester Step Test can predict maximal oxygen uptake (VO2max) [10]. Based on the value obtained in the Chester Step Test, participants were allocated to one of the three exercises programmes (light, moderate, or high intensity). All three exercise programmes had the same warm-up and cool-down exercises. The participants did the same exercise programme proposed at baseline during the ten-week intervention.

Instruction, monitoring, and feedback were realised by the m-health system including a smartphone (Motorola Moto G3) equipped with the EI app (movisensXS software, movisens GmbH, 2016), SD cards to store the exercise videos, and a mobile sensor (LightMove 3 wrist, movisens GmbH, 2016) to record physical activity and light exposure online throughout the intervention period (see Fig. S1). Participants were introduced to the usage of the app by trained study staff (clinicians or clinical researchers) and a user’s guide was handed over. Strengthening exercises were presented in the form of video sessions on the smartphones, which were executed while watching the videos. The videos presented an exercise specialist who carried out the different physical exercises proposed and subtitles showing graphical description, intensity, and rest of each exercise. The m-health app also allowed monitoring when participants started and stopped their aerobic and strengthening exercises; in addition, the EI was monitored by the activity sensor. The sensor was equipped with a Bluetooth Smart interface and offered the possibility of doing online analysis of data on the sensor. The sensor recorded the raw data of three-dimensional (3D) acceleration, barometric air pressure, and temperature. From these data, secondary parameters such as activity class, body position, steps, energy expenditure, and metabolic equivalents can be calculated with the movisens DataAnalyzer software. The sensor was fixed with a band at the wrist. Participants were asked to wear the sensor daily (24 h) during the ten-week intervention period. The m-health app also sent an acoustic signal to remind participants of their EI. An individual feedback (a reward summary including information on duration of the excises, movement acceleration intensity, and number of steps accompanied by a motivational message) was provided by the m-health app at the end of each day. Trained study staff had no access to this information but assessed participants’ intervention adherence via an interview at T3.


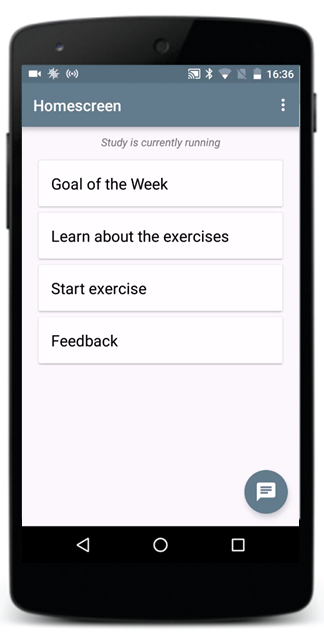

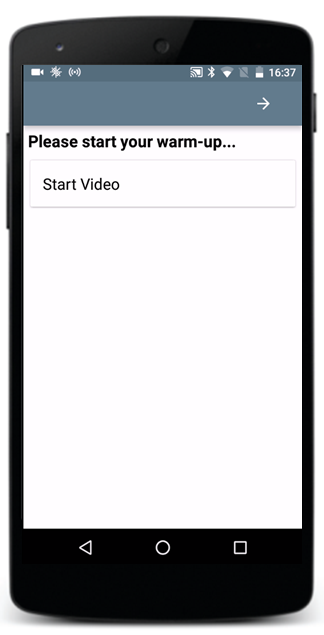

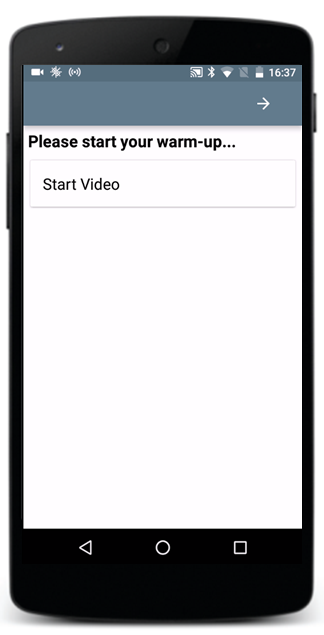

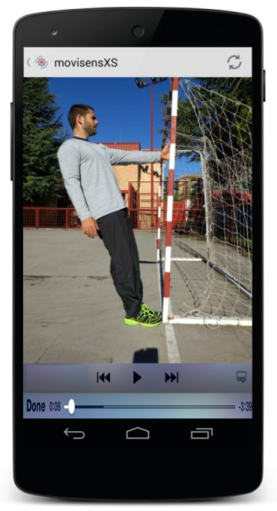

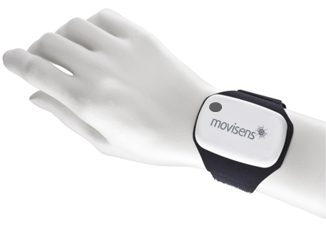

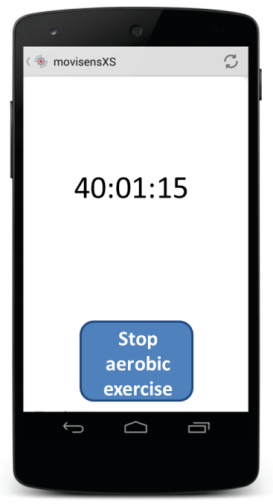

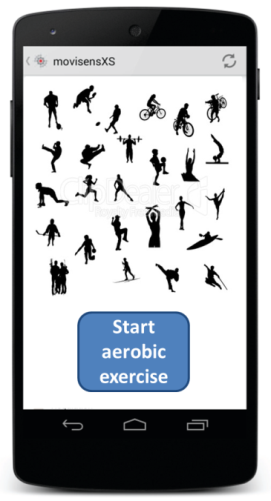


**Supplementary Fig. 1** M-Health system consisting of the smartphone and the sensor (adapted with permission of movisens) Example from the EI intervention. The “home screen” of the movisensXS app shows four different buttons: (1) Goal of the week; (2) Learn about the exercises; (3) Start exercise; (4) Feedback. If participants press button three (Start exercise), the exercise videos for the warm-up are played. If they choose aerobic activities, a timer is shown and they are asked to press the start button when they start exercises and the stop button when they finish exercises.

### **3.2 Bright light therapy (BLT)**

BLT consisted of a daily (except Sunday) 30-min exposure of white light without ultraviolet (UV) components in the morning or evening for ten weeks in total provided by special 10,000 lux light boxes that supply broadband, UV-filtered light, specifically designed for BLT (Philips EnergyLight HF 3419). The exact time of day of implementation (either during the morning between 06:00 and 08:00 am or the evening between 06:00 and 08:00 pm) was determined by the type of chronotype (evening type and morning type, respectively) of each study participant determined by the Morningness–Eveningness Questionnaire [11]. BLT was implemented in the morning for all participants except for two. The light therapy device was handed over by trained study staff (clinicians or clinical researchers) along with an introduction to the operation and how to carry out the light therapy at home. When receiving light therapy, participants sit approximately 50–75 cm from the light box, facing the illumination and glancing at the light occasionally. Participants were encouraged to read, watch TV, or work on a computer while the bright light was directed at their eyes.

Monitoring and feedback were realised with the m-health system comprising a smartphone (Motorola Moto G3) equipped with the BLT app (movisensXS software, movisens GmbH, 2016) and a mobile sensor (LightMove 3 wrist, movisens GmbH, 2016) to monitor the light exposure and physical activity of the participant (see Fig. S2). Participants were introduced to the usage of the m-health app by trained study staff (clinicians or clinical researchers) and a user’s guide was handed over. The m-health app allowed monitoring when participants started and stopped daily BLT. In addition, the light and activity sensor allowed us to monitor light exposure and activity (e.g., movement acceleration and number of steops) during the intervention period. Participants were asked to wear the LightMove 3 daily (24 h) fixed with a band at their wrist. The m-health app also sent an acoustic signal to remind participants of their BLT and provided them with individual feedback every day (i.e., a reward summary including information on duration of their BLT). Trained study staff had no access to this information but assessed participants’ intervention adherence via an interview at T3.


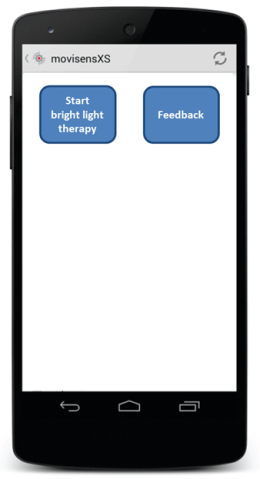

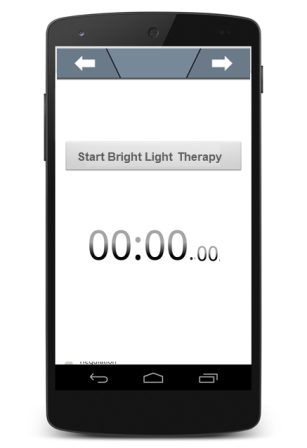

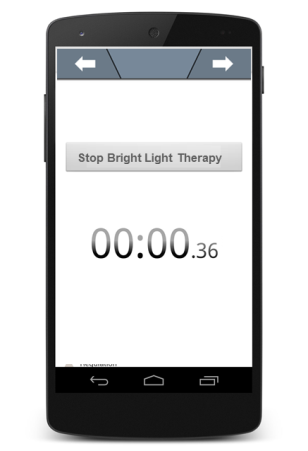

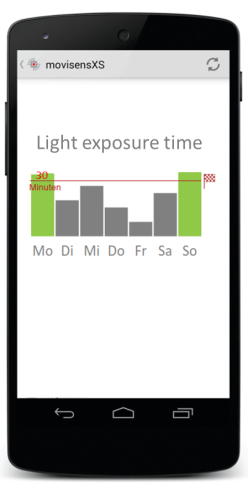

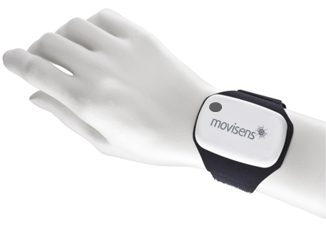


**Supplementary Fig. 2** M-Health system consisting of the smartphone and the sensor (adapted with permission of movisens) Example from the BLT. The “home screen” of the movisensXS app shows two different buttons: (1) Start BLT; (2) Feedback. If participants press start BLT, a timer is shown and they are asked to press the start button when they start the BLT session and to press the stop button when they finish the BLT session. Participants are provided with feeback in terms of the duration of their daily BLT (i.e., light exposure time) when they press the feedback button.

### **3.3 Treatment-As-Usual (TAU)**

Psychotropic medication that was started or changed at least four weeks before randomisation and that remained stable (mg/kg body weight) throughout the intervention and the three-month follow-up of the study (with the exception of dose adjustment to body weight changes) was allowed as TAU. The following psychotropic medication was allowed as single or combined treatment: any ADHD-specific medication, antidepressive treatment, and low dose neuroleptic treatment to control aggressive behaviour or mood swings. In addition, stable medication for the treatment of chronic conditions as allergies, asthma, enuresis, sleeping problems, and intermitting medication for acute infections or pain was allowed. Any individual-based (e.g. cognitive-behavioural therapy that does not include elements of BLT and EI, school-based intervention, occupational, language, psychomotor therapy) as well as family-based intervention that was started or changed at least four weeks before randomisation and that remained stable was allowed as TAU. Participants in the TAU group did not wear the sensor. Adherence to TAU was assessed via an interview at T3.

## **SI4. Study quality indicators**

### **4.1 Randomisation and masking procedures**

At each centre, the trial coordinator generated the randomisation code using a centralized web-based tool ([http://www.randomizer.at](http://www.randomizer.at/)) which was done at T2 after all baseline measurements had been completed in order to ensure allocation concealment. The randomisation list was kept in safe and confidential custody at the Institute of Medical Biometry and Informatics (IMBI). Participants withdrawn from the trial retained their identification codes (e.g. screening number, if already given). New participants were always allocated a new identification code. As the study was a non-pharmacological therapy intervention study, blinding of participants as well as study staff was not possible. However, the design was observer-blind. Trained clinicians/ clinical researchers, who assessed the primary efficacy outcome, the IDS-C_30_ total score, and the ADHD Rating Scale total score, were blind to treatment allocation. Therefore, at each site, randomisation was done only by the trial coordinator who was also the only person who managed the investigator site file (ISF). Trained clinicians/ clinical researchers who rated depressive and ADHD symptoms were not involved in the randomisation procedure and were not allowed to receive information about group assignment. They were provided with data recording sheets and data were entered into the CRF by the trial coordinator. In addition, participants were instructed not to talk about their intervention with the raters at each visit. Adherence to randomisation and masking procedures were monitored by an independent clinical on-site monitor, the Coordination Centre for Clinical Trials (KKS), University Hospital Heidelberg (see next paragraph).

### **4.2 Trial monitoring**

To ensure that the trial was conducted according to the protocol and regulatory requirements at all sites, the study was monitored by the the KKS, a university-based institution independent from other trial staff. Monitoring was done by on-site and off-site visits (e.g. at general assembly meetings) and frequent communication (letters, telephone, fascimile, email) by a clinical monitor according to standard operating procedures (SOPs) of the KKS and the study-specific monitoring manual, which outlined the trial procedures to be reviewed (e.g., filing and archiving of trial documents, source data verification, CRF documentation, adherence to protocol and regulatory requirements and observer-blinding requirements). Due to the COVID-19 pandemic, close-out visits were done remotely. The monitor ensured that the trial was conducted according to the protocol and regulatory requirements by review of source documents, entries into the CRFs, and essential documents. The monitor documented the visits in a report for the study PI as well as the local PI. The site was provided with a follow-up letter of the findings and the necessary actions to be taken. As the monitoring strategy considered current aspects of risk-based quality management, frequency of monitoring activities per site (up to four) varied depending on recruitment, experience, and general performance, e.g. quality of documentation of the individual trial sites. There were no major findings during monitoring. The study site at Nijmegen showed under-recruitment due to a delayed local start of the study. This was compensated by over-recruitment at the London and Frankfurt sites. As a result, recruitment needed to be extended by four months in total.

In addition to the standardised monitoring procedures, an independent Data Safety and Monitoring Board (DSMB) supervised the conduct of the trial and issued recommendations for early termination, modifications, or continuation of the trial, if necessary. The DSMB involved two independent clinical experts and one biometrician. The role of the DSMB was to monitor the progress of the trial and to ensure adherence to protocol. Serious adverse events (SAEs) were reported to the DSMB. The DSMB made recommendations for further actions to be taken and received follow-up reports.

### **4.3. Data management**

The IMBI Heidelberg was responsible for data management comprising all tasks concerning acquisition, processing, and utilisation of data with the aim of guaranteeing high quality of the data of this multicentre study and providing a valid data basis for the statistical analysis. The system used for data management was validated.

### **4.3.1 Data collection and transmission**

Questionnaire, test, and interview data as well as body parameters were sent regularly to the IMBI Heidelberg for data entry. The m-health sensor data were sent via “FileZilla – The free FTP solution” from all sites to the Karlsruhe Institute of Technology (KIT) and from the KIT to the IMBI Heidelberg by an AES-256 encryption. Therefore, all sites got their own protected and secure access to save and transfer data. Only the respective site and the KIT had the authority to enter this access. Afterwards, the data was transferred to the IMBI Heidelberg in the same protected way. In this case, only the KIT and the IMBI Heidelberg had the authority to enter this access.

Data was stored with pseudonyms only to protect participants. The security of the service was constantly checked by a security scan. The servers were hosted in a secure, ISO 27001 certified environment (datadock Strasbourg).

The app data on the smartphone was also encrypted (256 Bit). If a device got lost, remote reset was possible. All communication to the web console was highly encrypted with SSL. The data were decrypted as soon as it was in the secure web console. The security of the service was constantly checked by a security scan. The servers were hosted in a secure, ISO 27001 certified environment. Data was stored with pseudonyms only to protect participants. The app data was transferred from KIT to Heidelberg (IMBI) via “FileZilla” by an AES-256 encryption as well.

All findings including clinical data were documented in the participant’s medical record and in the case report form (CRF). The investigator was responsible for ensuring that all sections of the CRF were completed correctly and that entries could verified against source data (exception: questionnaire data was regarded as source data and part of the CRF at the same time). Any errors should have had a single line drawn through them so that the original entry remained legible and the correct data should have been entered at the side with the investigator’s signature, date, and reason for change. Self-explanatory corrections needed not to be justified. The completed CRF had to be reviewed and signed by the investigator named in the trial protocol or by a designated sub-investigator. The original CRF was transferred to the data management of the IMBI within three weeks after each participant trial visit (T2–T5); one copy remained with the investigator at the respective sites.

### **4.3.2 Data handling**

In order to ensure that the database reproduced the CRFs correctly, the IMBI accomplished a double entry of data (with the exception of free text) performed by two different persons. The completeness, validity, and plausibility of data were examined by validation programs, which thereby generated queries. The checks to be programmed were specified beforehand in a data validation plan. The investigator or the designated representatives were obliged to clarify or explain the queries. Any entry and correction in the study database was reported automatically in an audit file. If no further corrections were to be made in the database, it was closed (removal of write access) and used for statistical analysis. All data management activities were done according to the current SOPs of the IMBI.

**4.3.3 Storage and archiving of data**

The database server of the IMBI Heidelberg with the stored data was located in a secure environment and protected by a firewall. During the trial, the data access was restricted to data entry staff and the data manager responsible for the trial. After database closure, the biometrician responsible for the trial got access to the data for analysis. Backups were performed regularly.

The local investigators archived all trial data (participant identification code list, source data, and investigator’s file) and relevant correspondence in the ISF. The ISF was kept at each study site. Separate ISFs were provided for different adolescent and adult departments at the Goethe University, Frankfurt. At all other sites, trial data for adolescents and young adults were archived in one ISF. The ISF, all source data, and all documents indicated in section 8 of the ICH Consolidated Guideline on good clinical practice (GCP) (as applicable for the present study) was archived after finalisation of the trial according to the local legal regulations, at least for ten years. Trial-related documents were archived locally. The trial master file was archived at the Department of Child and Adolescent Psychiatry, Psychosomatics and Psychotherapy, Goethe University Hospital.

### **4.4 Training procedures and interrater reliability**

Group training sessions for all study staff (including clinicians and clinical researchers) involved in the trial from all centres took place at several consortium and kick-off meetings before the trial start. Trainings covered study requirements (e.g., observer-blind assessment of the primary outcome measure), general information about obtaining research quality data, and recording data (e.g. discussing data collection forms in detail on an item-by-item basis). Data collection forms and the SOPs could be downloaded from the CoCA intranet. The data to be collected and the procedures to be conducted at each visit were reviewed in detail. Furthermore, intervention manuals were discussed in detail at previous meetings and phone conferences. At subsequent CoCA consortium meetings and during regular monthly phone conferences, study staff were monitored with regard to the correct implementation of the manuals and evolving questions were discussed and solved. The training sessions also included training of the standardised procedures to assess primary and secondary outcome measures. Reliability training of the primary outcome measure was continued locally following a predefined training scheme (1. Observation of three interviews conducted by a trained clinician/ clinical researcher, 2. Rating of three video-taped interviews, 3. Conducting three interviews under supervision).

Interrater reliability ratings (IDS-C30; ADHD Rating Scale based on DIVA 2.0 or K-SADS) were based on a sample of video-taped interviews with participants assessed at KCL across visits (T2, T3, T4, T5). For the IDS-C30, 12 video-taped interviews (*n* = 2 adolescents, *n* = 10 adults) were rated by 11 clinicians/ clinical researchers from the different sites (Barcelona: *n* = 1, Frankfurt: *n* = 4, London: *n* = 4, Nijmegen *n* = 2). Of 132 potential ratings, 46 ratings were not conducted and ratings were missing. Numbers of rated interviews per rater are given in Supplementary Table 2. Intra-class correlation coefficient ICC(2,1)] together with its 95% confidence interval was calculated according to Shrout and Fleiss [12]. This intraclass correlation coefficient is based on a two-way analysis of variance and raters are considered as random effects measuring agreement between the judges. The analysis was conducted using R [13], version 4.0.2 and the package psych, version 2.0.9. The ICC(2,1) for the IDS-C_30_ was 0.93 (95% CI: 0.87-0.98) indicating good to excellent interrater reliability. For the ADHD interviews (DIVA 2.0, K-SADS), 11 video-taped interviews (*n* = 2 adolescents, *n* = 9 adults) were rated by 10 clinicians/ clinical researchers from all four sites (Barcelona: *n* = 1, Frankfurt: *n* = 4, London: *n* = 4, Nijmegen *n* = 1). Of 110 potential ratings, 34 ratings were not conducted and ratings are missing. Numbers of rated interviews per rater are given in Supplementary Table 2. The ICC(2,1) for the ADHD Rating Scale was 0.80 (95% CI: 0.63-0.93) indicating moderate to good interrater reliability.

### **Supplementary Table 2** Number of rated interviews per rater

| **Rater** | **IDS-C_30_** | **ADHD Rating Scale** |
| --- | --- | --- |
| #1 | 12 | 11 |
| #2  #3  #4  #5  #6  #7  #8  #9  #10  #11 | 4  12  10  4  4  3  12  12  1  12 | 2  11  9  4  4  3  11  10  NA  11 |

## **SI5. Feasibility outcomes**

The analysis followed a prespecified statistical analysis plan agreed upon by the principal investigator and the trial biometrician. Several feasibility outcomes were added (Amendment No.1 from 10/28/2021).

### **5.1 Feasibility outcomes in terms of the trial design**

- Number of screened participants
- Number of eligible individuals randomised to each group
- Number of participants who were randomised but did not start with intervention
- Number of drop-outs between T2 and T3, T3 and T4, and between T4 and T5
- Duration of recruitment

### **5.2 Feasibility outcomes in terms of data collection methods**

- Number of missing IDS-C_30_ scores per group at each visit (T2-T5)
- Number of missing BDI total scores per group at each visit (T2-T5)
- Number of missing ADHD Rating Scale total scores per group at each visit (T2-T5)
- Number of missing body composition parameters (BMI, body fat percentage, waist circumference, and waist-to-hip ratio) per group at each visit (T2-T5)
- Number of missing sensor data sets (mean light exposure, mean number of steps, and mean movement acceleration per day) at baseline (across 4 days of measurement between T1 and T2) (all groups)
- Number of missing sensor data sets (mean light exposure, mean number of steps, and mean movement acceleration per day) during 10-weeks of intervention (for BLT and EI groups only)
- Mean hours sensor on and worn per day at baseline (across 4 days of measurement between T1 and T2) (all groups)
- Number and percentage of observer-blind ratings (IDS-C_30_ and ADHD Rating Scale) per group at each visit (T2-T5)
- Interrater reliability for observer-based ratings of depression (IDS-C30) and ADHD symptoms (ADHD Rating Scale) (for details see 4.4)

### **5.3 Usability and acceptability of the m-health system (app and sensor)**

- Usability of the m-health app as rated by participants for baseline assessments (rated at T2, all groups) and for conducting BLT and EI (rated at T4, BLT and EI groups only) using the System Usability Scale (SUS). The SUS is a commonly used questionnaire for the evaluation of websites or mobile apps. The questionnaire consists of 10 items. Each item represents a statement (e.g., “I thought the system was easy to use.”) and participants rate their agreement with the statement on a 5-point scale. A total score between 0 and 100 is calculated whereas higher numbers indicated better usability (SUS scores of > 70 indicate acceptable usability, whereas SUS scores between 50 and 70 indicate marginal acceptibility) [2].
- Percentage wear time of sensor (sensor on and worn) during 10 weeks of intervention (for BLT and EI groups only)
- Mean hours sensor on and worn during 10 weeks of intervention (for BLT and EI groups only)

### **5.4 Participants’ adherence to interventions**

- Percentage of conducted BLT/ EI sessions as indicated by self-reports recorded online with the m-health app
- Total duration of BLT during 10 weeks of intervention (in minutes) as indicated by self-reports recorded online with the m-health app
- Mean duration of one BLT session (in minutes) as indicated by self-reports recorded online with the m-health app
- Total duration of aerobic EI during 10 weeks of intervention (in minutes) as indicated by self-reports recorded online with the m-health app
- Mean duration of one aerobic EI session (in minutes) as indicated by self-reports recorded online with the m-health app
- Mean light exposure per day during wake time (in lux) during 10 weeks of intervention as measured with the sensor (BLT and EI groups only)
- Physical activity per day during wake time (mean number of steps and movement acceleration) during 10 weeks of intervention as measured with the sensor (BLT and EI groups only)
- Adherence to BLT, EI, and TAU during the first 5 weeks of intervention as assessed retrospectively via an interview at T3 (adherence given in case >= 80% of sessions were done (BLT/ EI))
- Change in type and dose of prescribed concomitant medication and change in type and frequency of concomitant psychosocial treatment recorded at each visit (T2-T5) (absolute/ relative frequencies; percentage, missings)
- Acceptibility of treatments: participants’ self-reports on continuation with BLT and EI after T4 (assessed via an interview at T5): number of participants who continued with intervention after T4 and frequency of BLT/ EI sessions for participants who continued

### **5.5 Treatment integrity**

Study staffs’ adherence in delivering the prescribed treatment protocol:

- procedures of introducing the intervention and the usage of the m-health system according to the SOP at T2 (absolute/ relative frequencies; percentage, missings)
- conducting motivational interviews at T3 in case of low adherence to the interventions (absolute/ relative frequencies; percentage, missings)

## **SI6. Assessment of adverse and serious adverse events**

Adverse events (AEs) and serious adverse events (SAEs) were assessed in all randomised participants. (S)AEs were classified with regard to intensity (mild, moderate, severe), causality with intervention (certain, probable, possible, unlikely, unrelated, not assessed), and action with intervention (unchanged, reduced, increased, discharged, unknown, not applicable), and were followed up by the local investigator (countermeasures and outcomes were monitored). All SAEs were reported to the principal investigator, the clinical monitor, and the DSMB. The DSMB made recommendations for further actions to be taken and received follow-up reports.

### **Supplementary Table 3** Adverse events in the safety analysis set (all randomised participants) by received intervention

|  | **TAU *n* = 68** | **BLT  *n* = 70** | **EI *n* = 69** | **Total *n* = 207** |
| --- | --- | --- | --- | --- |
|  |  |  |  |  |
| Total no. of AEs | 64 | 62 | 55 | 181 |
| Min no. of AEs per patient  Max no. of AEs per patient  Mean no. of AEs per patient  No. of participants with at least one AE | 0  6  0.9 (1.4)  29 (42.6%) | 0  5  0.9 (1.1)  39 (55.7%) | 0  5  0.8 (1.2)  31 (44.9%) | 0  6  0.9 (1.2)  99 (47.8%) |
| Causality with intervention per patient |  |  |  |  |
| - No AE | 39 (57.4%) | 31 (44.3%) | 38 (55.1%) | 108 (52.2%) |
| - certain | 0 (0.0%) | 2 (2.9%) | 0 (0.0%) | 2 (1.0%) |
| - probable | 0 (0.0%) | 1 (1.4%) | 1 (1.4%) | 2 (1.0%) |
| - possible | 0 (0.0%) | 0 (0.0%) | 2 (2.9%) | 2 (1.0%) |
| - unlikely | 0 (0.0%) | 1 (1.4%) | 3 (4.3%) | 4 (1.9%) |
| - unrelated | 26 (38.2%) | 35 (50.0%) | 25 (36.2%) | 86 (41.5%) |
| - not assessed | 3 (4.4%) | 0 (0.0%) | 0 (0.0%) | 3 (1.4%) |
|  |  |  |  |  |

AE = adverse event, BLT = bright light therapy, EI = exercise intervention, no. = number, TAU = treatment as usual.

### **Supplementary Table 4** Serious adverse events in the safety analysis set (all randomised participants) by received intervention

|  | **TAU *n* = 68** | **BLT *n* = 70** | **EI *n* = 69** | **Total *n* = 207** |
| --- | --- | --- | --- | --- |
|  |  |  |  |  |
| Total no. of SAEs | 2 | 1 | 4 | 7 |
| Min no. of SAEs per patient  Max no. of SAEs per patient  Mean no. of SAEs per patient  No. of participants with at least one SAE | 0  1  0.0 (0.2)  2 (2.9%) | 0  1  0.0 (0.1)  1 (1.4%) | 0  2  0.1 (0.3)  3 (4.3%) | 0  2  0.0 (0.2)  6 (2.9%) |
| Causality with intervention per patient |  |  |  |  |
| - No SAE | 66 (97.1%) | 69 (98.8%) | 66 (95.7%) | 201 (97.1%) |
| - unlikely | 0 (0.0%) | 0 (0.0%) | 1 (1.4%) | 1 (0.5%) |
| - unrelated | 2 (2.9%) | 1 (1.4%) | 2 (2.9%) | 5 (2.4%) |
|  |  |  |  |  |

SAE = serious adverse event, BLT = bright light therapy, EI = exercise intervention, no. = number, TAU = treatment as usual.

## **SI7. Efficacy outcomes**

### **7.1 Primary efficacy outcome**

The primary efficacy outcome was the change in the observer-rated IDS-C_30_ total score (observer-blind assessment) between T2 and T4. The IDS-C_30_ rating includes all DSM-5 diagnostic criterion items for major depressive disorder (e.g., mood, vegetative, psychomotor, and cognitive symptoms) as well as commonly associated symptoms such as anxiety, irritability, melancholic, and atypical symptom features to assess the severity of depressive symptoms over the last seven days. Items are rated on a 4-point Likert scale based on the information obtained during a semi-structured interview. The total score range is 0 to 84. The psychometric properties of the IDS-C_30_ and its sensitivity to change with interventions have been well established in different study samples and RCTs [14–16]. Parallel versions exist in English, German, Spanish, and Dutch. The IDS is under investigation in adolescent patients, however a standardisation for individuals between 14 and 17 years of age is still lacking. Because changes in raw scores were assessed in this study, the IDS-C_30_ was considered a valid measure in adolescents.

### **7.2 Secondary efficacy outcomes**

The following secondary outcome measures were assessed at baseline, directly after the intervention/ continuing TAU (T4) and/ or at 12-weeks follow-up (T5):

- Change in the IDS-C_30_ total score (observer-blind assessment) between T2 and T5
- Change in the Beck Depression Inventory (BDI-II) total summary score between T2 and T4 and between T2 and T5: The BDI-II is widely used self-report scale for persons age 13 and older to assess the severity of depressive symptoms [17]. It contains 21 items that are rated on a Likert (0-3 coded) scale.
- Change in ADHD symptom severity total score and subscale scores as assessed by the ADHD Rating Scale between T2 and T4 and between T2 and T5: Separate versions exist for children/ adolescents [18] and adults [19]. Both scales are exactly comparable 18-item scales assessing ADHD symptoms (adjusted to DSM-5 criteria) by a 4-point Likert-type severity scale. The scales give a total ADHD summary score (items 1-18), as well as separate summary scores for inattentive (items 1-9), hyperactive/ impulsive (items 10-18) subscales. The observer-rated symptom severity for each item was based on his or her interview with the adult participant based on the DIVA [4]. For adolescents, symptom severity was rated for each item based on the K-SADS-PL [3]. Ratings were done observer-blind.
- Change in body composition parameters between T2 and T4 and between T2 and T5: Change in Body Mass Index (BMI), waist circumference (in cm), waist-to-hip ratio, and body fat percentage calculated based on skinfold thickness measurements using a skinfold caliper (Holtain Skinfold Caliper). Body fat percentage (BF) was calculated using the formula proposed by [20]: BF = (4.57/BD – 4.142) x 100, where BD is body density. Body density is calculated using the formula BD = 1.1765 - 0.0744 (log_10_X) for men and BD = 1.1567 – 0.0717(log_10_X) for women, where X is the sum of the following skinfold thicknesses in mm: triceps + biceps + subscapular + supraspinale. Measurements were not done observer-blind.

## **SI8. Statistical analyses**

In the statistical analysis plan, no imputation strategy for baseline covariates was described. Because there were five missing values in the baseline IQ, missing values were imputed. Therefore, a single imputation regression approach was used with centre, treatment group, age, gender and baseline IDS-C_30_ as covariates. Regarding missing values in the primary efficacy endpoint, the mITT set was defined so that at least one follow-up IDS-C_30_ total score at T3 or T4 was available. If a missing value occurred at T4, the applied mixed model for repeated measures (MMRM) model compensated for a missing value at T4 and the patient was still considered in the analysis. The MMRM approach modeled jointly all actual observations without imputing missing data but using the within-participant correlation structure to provide information about unobserved post-baseline primary endpoint. All analyses were performed using SAS® Software version 9.4 (SAS Inc., Cary/NC, USA). Sample size calculation was done using ADDPLAN v6.1.

Sensitivity analyses included a per-protocol analysis, a complete case analysis and applying different imputation techniques as last-observation-carried-forward (LOCF) for missing values.

The per-protocol population included patients without major protocol violations. Major protocol violations were defined in terms of violating inclusion or exclusion criteria, a mean adherence to the intervention across 10 weeks of intervention (BLT or EI, measured via the smartphone app) of less than 80% of conducted sessions, and/ or a T4 visit that deviates more than +/- 7 days from the date planned according to the protocol. Due to the rather low adherence to either EI and BLT, the sample size in the EI and BLT group of the per-protocol set was very small. Hence, in addition to the analysis of the presepecified per-protocol set, the analysis was conducted for two additional populations defined by the following criteria:

• Modified per-protocol set – version 1: A more generous threshold value of 50% (instead of 80%) for the adherence was used. All other criteria for the per-protocol set remained unchanged.

• Modified per-protocol set – version 2: A more generous threshold value of 50% for the adherence was used and the participants with a deviation of more than ± 7 days from the date planned according to the protocol were not excluded. As in the mITT set the IDS-C_30_ total score had to be available at T2 and at T3 and/ or T4.

The complete case analysis was conducted for participants whose IDS-C_30_ total score was not missing at T2 and T4. According to the SAP, IDS-C_30_ total score was missing if 25 or less items were available. An analysis of covariance was used instead of the MMRM model since observations at T4 were complete. The covariates baseline IDS-C30, age, and IQ and the cofactors gender, treatment, and centre were included.

As imputation technique for missing values LOCF was also used. If the IDS-C_30_ total score was missing at T4, it was replaced by the score at T3. For this analysis, the analysis of covariance was used as described for the complete case analysis.

The effect of adherence to BLT/ EI (as percentage of conducted sessions as measured online by the m-health app) during the 10 weeks treatment period was investigated using adherence as a further covariate in the MMRM model described above. Since adherence was only measured for the two intervention groups, the TAU group was not considered in this analysis. 105 participants were included in this analysis (54 in BLT group and 51 in EI). Adherence data was missing for 5 participants in BLT group and 3 participants in the EI group.

**Supplementary Table 5** Number of screened and randomised participants per centre

| **Centre** |  | | **screened** | **randomised** |
| --- | --- | --- | --- | --- |
| Goethe University Hospital Frankfurt  King’s College London  Radboud University Medical Centre, Nijmegen  Vall d’Hebron Research Institute, Barcelona  Total | |  | 229  84  136  104  553 | 69 (30%)  59 (70%)  29 (21%)  50 (48%)  207 (37%) |

Data are *n* (%).

**Supplementary Table 6** Demographic and clinical characteristics at baseline for participants in the mITT population

|  | | **TAU (*n* = 61)** | **BLT (*n* = 59)** | **EI (*n* = 54)** |
| --- | --- | --- | --- | --- |
| Sex  Male  Female  Age (years), mean (range)  IQ^a^  Highest education of patient^b^  Highest education of parents^c^  Dependent on welfare^d^  Current school/apprenticeship/college/job attendance  Full-time  Part-time  ADHD subtype  Combined  Predominantly inattentive  Predominantly hyperactive-impulsive  At least one comorbid psychiatric disorder  Depressive disorders  Anxiety disorders  Obsessive-compulsive and tic disorders  Externalising disorders  Chronic medical problems^e^  At least one concomitant medication  Stimulants  Non-stimulant ADHD medication  Anti-depressive medication  Antipsychotic medication  Concomitant psychosocial treatment^f^  IDS-C_30_ total score, mean (*SD*, *n* missing data)  BDI total score, mean (*SD*, *n* missing data)  ADHD Rating scale, mean (*SD*, *n* missing data)  Total  Inattentive subscale  Hyperactive/ impulsive subscale  BMI (kg/m^2^), mean (*SD*, *n* missing data)  Obesity Class I  Obesity Class II  Obesity Class III  Waist circumference (cm), mean (*SD*, *n* missing data)  Waist-to-hip ratio, mean (*SD*, *n* missing data)  Body fat percentage, mean (*SD*, *n* missing data)  Mean number of steps per day (*SD*, n missing data)  Mean movement acceleration per day (*SD*, *n* missing data)  Mean light exposure per day (*SD*, *n* missing data) | 33 (54%)  28 (46%)  25 (14-44)  106 (13.1)  3.5 (1.1)  3.7 (0.8)  7 (12%)  32 (53%)  13 (21%)  43 (71%)  17 (28%)  1 (2%)  19 (31%)  12 (20%)  5 (8%)  1 (2%)  2 (3%)  9 (15%)  49 (80%)  43 (71%)  3 (5%)  8 (13%)  2 (3%)  11 (18%)  15.1 (11.0, 0)  12.9 (10.2, 1)  27.1 (9.2, 1)  15.3 (5.0, 0)  11.9 (5.6, 1)  24.3 (5.1, 1)  4 (7%)  2 (3%)  0  80.9 (14.3, 0)  0.8 (0.1, 0)  25.7 (7.4, 0)  15686 (5986, 15)  157.1 (44.7, 15)  396.6 (479.5, 15) | | 23 (39%)  36 (61%)  26 (14-44)  107 (12.5)  3.8 (1.1)  4.0 (1.1)  6 (10%)  30 (51%)  10 (17%)  37 (64%)  20 (35%)  1 (2%)  13 (22%)  8 (14%)  3 (5%)  1 (2%)  0 (0%)  17 (29%)  46 (78%)  38 (64%)  4 (7%)  12 (20%)  1 (2%)  5 (9%)  13.5 (7.7, 0)  14.4 (10.2, 0)  27.2 (9.0, 0)  15.6 (4.8, 0)  11.6 (5.7, 0)  25.0 (5.2, 1)  5 (9%)  3 (5%)  1 (2%)  82.0 (16.2, 2)  0.8 (0.1, 2)  27.6 (6.7, 1)  13842 (5030, 9)  148.1 (33.7, 9)  341.7 (391.8, 9) | 24 (44%)  30 (56%)  26 (14-44)  108 (12.4)  3.7 (1.2)  3.8 (1.3)  7 (13%)  31 (57%)  8 (15%)  34 (63%)  19 (35%)  1 (2%)  14 (26%)  8 (15%)  2 (4%)  1 (2%)  1 (2%)  14 (27%)  47 (87%)  42 (78%)  3 (6%)  10 (19%)  2 (4%)  9 (17%)  13.2 (8.1, 0)  12.6 (9.5, 2)  24.5 (8.4, 0)  14.8 (5.4, 0)  9.7 (5.0, 0)  25.8 (6.2, 0)  4 (7%)  5 (9%)  2 (4%)  85.9 (15.7, 0)  0.8 (0.1, 0)  27.2 (7.8, 0)  15274 (5805, 12)  148.9 (35.6, 12)  466.3 (526.0, 13) |

*Note.* Data are *n* (%) or mean (*SD*), unless otherwise specified. ^a^Verbal and nonverbal intelligence were estimated by the vocabulary and matrix reasoning subtests of WAIS-IV[8] in adults and the WISC-IV [9] in adolescents. The mean IQ calculated across both tasks is reported. ^b^Numeric education status was calculated as follows: ISCED 0 = 0: pre-primary, ISCED 1 = 1: primary, ISCED 2A = 2: lower secondary, ISCED 3A, B, C = 3: upper secondary, ISCED 4A = 4: post secondary, ISCED 5 A, B = 5: lower tertiary, ISCED 6 = 6: higher tertiary education. ^c^Parental education status represents the mean of the biological father’s and mother’s ISCED score. If data for one biological parent was missing, the other biological parent’s score was used. ^d^Dependence on welfare (yes/ no) was assessed in order to define socioeconomic status. ^e^Chronic medical problems included for example asthma, hypothyroidism, allergies, headaches/ migraine, back pain, and hypertension. ^f^Concomitant psychosocial treatment included psychotherapy (individual or group based), language therapy, and family based interventions. BDI-II = Beck Depression Inventory, 2nd version, BLT = bright light therapy, BMI = body mass index, EI = exercise intervention, IDS-C_30_ = Inventory of Depressive Symptomatology, ISCED = International Standard Classification of Education, TAU = treatment as usual. Mean number of steps, mean movement acceleration, and mean light exposure were calculated for wake time.

**Supplementary Table 7** Number of missing data for assessment of depressive and ADHD symptom severity at each visit for all randomised participants

|  | **Baseline** | **T3** | **T4** | **T5** |
| --- | --- | --- | --- | --- |
| **IDS-C_30_** |  |  |  |  |
| Total (*n* = 207)  TAU (*n* = 68)  BLT (*n* = 70)  EI (*n* = 69) | 5  1  2  2 | 40 (29)  10 (6)  15 (9)  15 (14) | 45 (42)  9 (9)  17 (16)  19 (17) | 63 (60)  19 (17)  21 (21)  23 (22) |
|  |  |  |  |  |
| **BDI-II**  Total (*n* = 207)  TAU (*n* = 68)  BLT (*n* = 70)  EI (*n* = 69)  **ADHD Rating Scale Total Score** | 6  1  1  4 | 45 (29)  13 (6)  14 (9)  18 (14) | 52 (42)  12 (9)  21 (16)  19 (17) | 64 (60)  20 (17)  21 (21)  23 (22) |
| Total (*n* = 207)  TAU (*n* = 68)  BLT (*n* = 70)  EI (*n* = 69) | 3  1  1  1 | 40 (29)  10 (6)  15 (9)  15 (14) | 46 (42)  10 (9)  18 (16)  18 (17) | 62 (60)  19 (17)  21 (21)  22 (22) |

Missing data because of premature study termination are included. Number of drop-outs are given in parenthesis.

**Supplementary Table 8** Number of missing data for assessment of body composition parameters at each visit for all randomised participants

|  | **Baseline** | **T3** | **T4** | **T5** |
| --- | --- | --- | --- | --- |
| **BMI** |  |  |  |  |
| Total (*n* = 207)  TAU (*n* = 68)  BLT (*n* = 70)  EI (*n* = 69) | 5  1  3  1 | 50 (29)  13 (6)  17 (9)  20 (14) | 65 (42)  17 (9)  24 (16)  24 (17) | 90 (60)  30 (17)  31 (21)  29 (22) |
| **Body fat percentage** |  |  |  |  |
| Total (*n* = 207) | 5 | 51 (29) | 70 (42) | 90 (60) |
| TAU (*n* = 68)  BLT (*n* = 70)  EI (*n* = 69) | 0  4  1 | 13 (6)  18 (9)  20 (14) | 18 (9)  27 (16)  25 (17) | 31 (17)  30 (21)  29 (22) |
|  |  |  |  |  |
| **Waist circumference** |  |  |  |  |
| Total (*n* = 207)  TAU (*n* = 68)  BLT (*n* = 70)  EI (*n* = 69) | 6  1  4  1 | 52 (29)  13 (6)  19 (9)  20 (14) | 67 (42)  18 (9)  25 (16)  24 (17) | 89 (60)  30 (17)  30 (21)  29 (22) |
|  |  |  |  |  |
| **Waist-to-hip ratio** |  |  |  |  |
| Total (*n* = 207)  TAU (*n* = 68)  BLT (*n* = 70)  EI (*n* = 69) | 6  1  4  1 | 52 (29)  13 (6)  19 (9)  20 (14) | 67 (42)  18 (9)  25 (16)  24 (17) | 89 (60)  30 (17)  30 (21)  29 (22) |

Missing data because of premature study termination are included. Number of drop-outs are given in parenthesis.

**Supplementary Table 9** Mean wearing time of the light and activity sensor at baseline for all randomised participants

|  | **TAU *n* = 68** | **BLT *n* = 70** | **EI *n* = 69** | **Total *n* = 207** |
| --- | --- | --- | --- | --- |
| Mean hours sensor on and worn per day |  |  |  |  |
| - *n* | 55 | 59 | 55 | 169 |
| - Mean +/- SD | 17.4 +/-6.8 | 17.7 +/-5.5 | 18.6 +/-5.9 | 17.9 +/-6.1 |
| - Median | 20.3 | 19.7 | 21.0 | 20.0 |
| - p25, p75 | 15.0, 22.4 | 15.7, 21.5 | 17.6, 22.5 | 15.9, 22.2 |
| - Min, Max | 0.0, 23.8 | 0.0, 23.7 | 0.0, 23.9 | 0.0, 23.9 |
| - Missing^a^ | 13 (19 %) | 11 (16 %) | 14 (20 %) | 38 (18 %) |

^a^Number and percentage of participants without valid m-health data.

**Supplementary Table 10** Mean wearing time of the light and activity sensor during 10 weeks of intervention for participants who received the intervention

|  | | **BLT *n* = 69** | **EI *n* = 68** |
| --- | --- | --- | --- |
| Mean % wearing time of sensor (sensor on and worn) during 10 weeks of intervention | - *n*  - Mean +/- SD  - Median  - p25, p75  - Min, Max  - Missing^a^ | 63  30.1 +/-28.1  19.4  6.4, 57.9  0.0, 93.7  6 (9 %) | 59  41.4 +/-29.6  40.7  11.4, 69.6  0.1, 91.6  9 (13%) |
| Mean hours sensor on and worn during 10 weeks of intervention | - *n*  - Mean +/- SD  - Median  - p25, p75  - Min, Max  - Missing^a^ | 63  512.4 +/-478.6  330.7  109.0, 986.5  0.0, 1596.2  6 (9 %) | 59  695.7 +/-498.0  683.3  191.5, 1169.2  2.1, 1539.3  9 (13%) |

^a^Number and percentage of participants without valid m-health data.

**Supplementary Table 11.** Mean System Usability Scale (SUS) score at T4 for participants

who received the intervention

|  | | **BLT *n* = 69** | **EI *n* = 68** |
| --- | --- | --- | --- |
| Mean SUS score assessed at T4 | - *n*  - Mean +/- SD  - Median  - p25, p75  - Min, Max  - Missing | 49  76.1 +/-14.4  77.5  70.0, 85.0  35.0, 100.0  20 (30 %) | 46  65.5 +/-18.7  63.8  55.0, 82.5  22.5, 100.0  22 (32%) |

**Supplementary Table 12** Adherence to BLT

|  | | **BLT *n* = 69** |
| --- | --- | --- |
| Mean percentage of conducted BLT sessions over 10 weeks | - *n*  - Mean +/- SD  - Median  - p25, p75  - Min, Max  - Missing | 60  52.5 +/-30.4  50.8  27.5, 74.2  1.7, 113.3  9 |
| Categorised adherence to BLT | - < 10%  - 10% - 20%  - 20% - 30%  - 30% - 40%  - 40% - 50%  - 50% - 60%  - 60% - 70%  - 70% - 80%  - 80% - 90%  - >= 90% | 5 (8.3%)  4 (6.7%)  6 (10.%)  5 (8.3%)  9 (15.0%)  7 (11.7%)  4 (6.7%)  7 (11.7%)  4 (6.7%)  9 (15.0%) |
|  |  |  |
| Mean duration of each BLT session (in minutes)^a^ | - *n*  - Mean +/- SD  - Median  - p25, p75  - Min, Max  - Missing | 60  31.1 +/-5.3  31.1  29.8, 32.7  16.7, 56.2  9 |
| Mean total duration of BLT over 10 weeks (in minutes) | - *n*  - Mean +/- SD  - Median  - p25, p75  - Min, Max  - Missing | 61  960.4 +/-617.5  860.9  433.9, 1413.0  0.0, 2572.0  8 |
| Total duration of BLT during 10 weeks of intervention (in minutes)/ total duration of BLT planned (1800 min) | - *n*  - Mean +/- SD  - Median  - p25, p75  - Min, Max  - Missing | 61  0.5 +/-0.3  0.5  0.2, 0.8  0.0, 1.4  8 |

^a^Values higher than 60 minutes were set to target time (30 minutes).

**Supplementary Table 13** Physical activity and light exposure during 10 weeks of intervention for participants who received the intervention^a^

|  | | **BLT *n* = 69** | **EI *n* = 68** |
| --- | --- | --- | --- |
| Mean number of steps per day | - *n*  - Mean +/- SD  - Median  - p25, p75  - Min, Max  -Missing | 23  11666.7 +/-3937.7  10943.7  8697.7, 15025.9  5042.3, 19203.4  46 (67%) | 32  14788.5 +/-6032.5  14066.8  10447.2, 17055.0  4802.1, 30644.0  36 (53%) |
| Mean movement acceleration per day | - *n*  - Mean +/- SD  - Median  - p25,p75  - Min, Max  - Missing | 23  137.3 +/-30.7  129.6  118.3, 165.6  76.9, 213.5  46 (67%) | 32  159.0 +/-37.2  162.0  134.3, 184.0  87.2, 237.6  36 (53%) |
|  |  |  |  |
| Mean light exposure per day in lux | - *n*  - Mean +/- SD  - Median  - p25, p75  - Min, Max  - Missing | 21  336.5 +/-314.7  238.1  80.4, 505.3  33.3, 1133.6  48 (70%) | 30  392.9 +/-392.0  264.5  121.0, 517.6  60.3, 1665.6  38 (56%) |

^a^Physical activity and light exposure per day were calculated for time awake. Participants who wore the sensor less than 8 hours per day were excluded from the analyses.

**Supplementary Table 14** Adherence to EI

|  | | **EI *n* = 68** |
| --- | --- | --- |
| Mean percentage of conducted EI sessions over 10 weeks | - *n*  - Mean +/- SD  - Median  - p25, p75  - Min, Max  - Missing | 59  27.6 +/-28.3  15.8  7.5, 40.0  0.0, 115.0  9 |
| Categorised adherence to EI | - < 10%  - 10% - 20%  - 20% - 30%  - 30% - 40%  - 40% - 50%  - 50% - 60%  - 60% - 70%  - 70% - 80%  - 80% - 90%  - >= 90% | 18 (30.5%)  14 (23.7%)  5 (8.5%)  7 (11.9%)  2 (3.4%)  4 (6.8%)  4 (6.8%)  1 (1.7%)  1 (1.7%)  3 (5.1%) |
|  |  |  |
| Mean percentage of conducted strenghtening exercise sessions over 10 weeks | - *n*  - Mean +/- SD  - Median  - p25, p75  - Min, Max  - Missing | 59  23.9 +/-30.3  10.0  5.0, 40.0  0.0, 120.0  9 |
|  |  |  |
| Mean percentage of conducted aerobic excercise sessions over 10 weeks | - *n*  - Mean +/- SD  - Median  - p25, p75  - Min, Max  -Missing | 59  31.2 +/-28.4  20.0  6.7, 53.3  0.0, 110.0  9 |
| Mean duration of each aerobic exercise session (in minutes) | - *n*  - Mean +/- SD  - Median  - p25, p75  - Min, Max  - Missing | 49  32.0 +/-14.4  31.4  23.7, 39.3  0.1, 63.1  19 |
| Mean total duration of aerobic exercise sessions over 10 weeks (in minutes) | - *n*  - Mean +/- SD  - Median  - p25, p75  - Min, Max  - Missing | 54  354.8 +/-331.6  256.1  64.5, 544.8  0.0, 1259.0  14 |
| Total duration of aerobic exercise sessions during 10 weeks (in minutes)/ total duration planned (230/ 340 min) | - *n*  - Mean +/- SD  - Median  - p25, p75  - Min, Max  - Missing | 54  1.2 +/-1.1  0.8  0.3, 1.9  0.0, 3.7  14 |

**Supplementary Table 15** Changes in type and dose/ freqency of concomitant medication and psychosocial treatment during study participation

|  | **TAU *n* = 68** | **BLT *n* = 69** | **EI *n* = 68** | **Total *n* = 205** |
| --- | --- | --- | --- | --- |
| Change in type of medication between |  |  |  |  |
| - T2 and T3 | 7 | 5 | 5 | 17 |
| - T3 and T4 | 1 | 6 | 2 | 9 |
| - T4 and T5  Total | 4  12 (18%) | 4  15 (22%) | 5  12 (18%) | 13  39 (19%) |
| Change in dose of medication between |  |  |  |  |
| - T2 and T3 | 3 | 1 | 4 | 8 |
| - T3 and T4 | 4 | 2 | 0 | 6 |
| - T4 and T5  Total | 3  10 (15%) | 6  9 (13%) | 2  6 (9%) | 11  25 (12%) |
| Change in type of psychsocial treatment between  - T2 and T3  - T3 and T4  - T4 and T5  Total  Change in frequency of psychsocial treatment between  - T2 and T3  - T3 and T4  - T4 and T5  Total | 0  1  1  2 (3%)  0  0  1  1 (1.5%) | 0  1  1  2 (3%)  0  0  0  0 | 0  0  0  0 (0%)  1  0  0  1 (1.5%) | 0  2  2  4 (2%)  1  0  1  2 (1%) |

Data are *n* and % of randomised participants who started their intervention/ TAU.

**
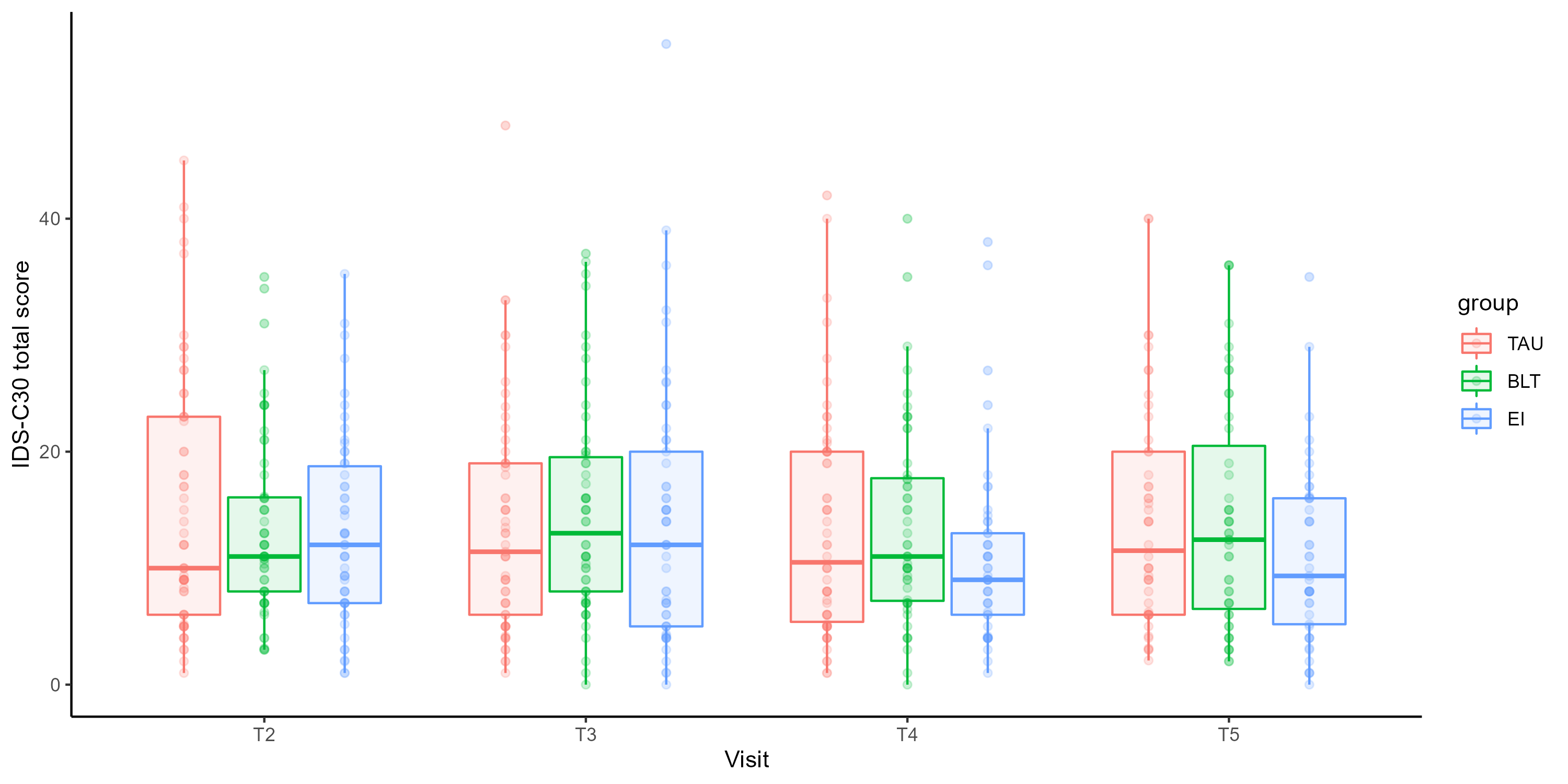
**

**Supplementary Fig. 3** Mean IDS-C_30_ total scores by randomised group at each visit (T2-T5) for the 174 participants of the mITT set. BLT, bright light therapy; EI, exercise intervention; IDS-C_30_, Inventory of Depressive Symptomatology; mITT, modified intention-to-treat set; TAU, treatment-as-usual.


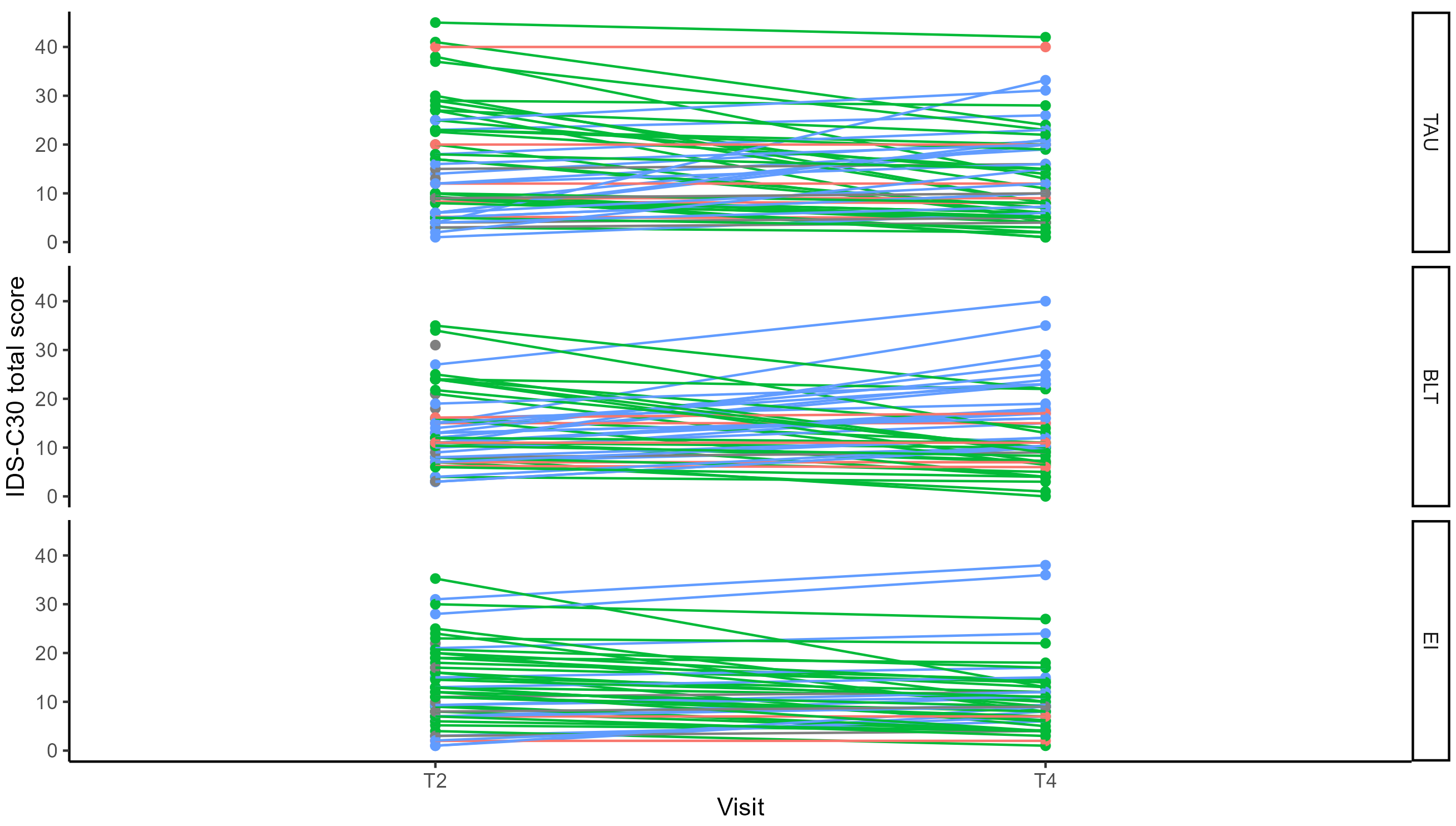

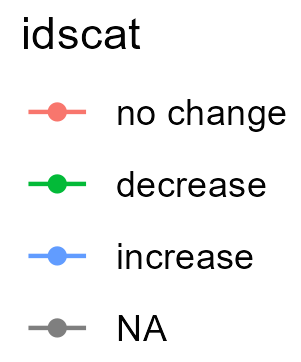


**Supplementary Fig. 4** Changes in IDS-C_30_ total scores from baseline to T4 for each participant of the mITT set.

BLT, bright light therapy; EI, exercise intervention; idscat, category of type of change from baseline (T2) to T4 (post-intervention) in IDS-C_30_ total scores (no change, decrease, increase; NA, not applicable); IDS-C_30_, Inventory of Depressive Symptomatology; mITT, modified intention-to-treat set; TAU, treatment-as-usual

**Supplementary Table 16** Summary measures (without imputed values) for the primary outcome (IDS-C_30_ total score, difference T4-T2) in the different per-protocol (PP) sets

| **PP set** | **TAU *n* = 43** | **BLT *n* = 10** | **EI *n* = 4** | **Total *n* = 57** |
| --- | --- | --- | --- | --- |
| Mean +/- SD  Median  P25, p75  Min, Max | -1.5 +/-9.6  -1.0  -6.0, 3.3  -25.0, 29.2 | 3.0 +/-7.6  4.5  0.0, 7.0  -15.0, 11.9 | -5.3 +/-7.1  -4.0  -9.5, -1.0  -15.0, 2.0 | -0.9 +/-9.2  -0.2  -4.0, 5.0  -25.0, 29.2 |

| **Additional PP set – version 1** | **TAU *n* = 43** | **BLT *n* = 23** | **EI *n* = 10** | **Total *n* = 76** |
| --- | --- | --- | --- | --- |
| Mean +/- SD  Median  P25, p75  Min, Max | -1.5 +/-9.6  -1.0  -6.0, 3.3  -25.0, 29.2 | 2.3 +/-7.8  4.0  -1.0, 7.0  -15.7, 14.0 | -2.9 +/-5.9  -3.7  -5.3, 1.0  -15.0, 7.0 | -0.5 +/-8.7  0.0  -4.0, 5.0  -25.0, 29.2 |

| **Additional PP set – version 2** | **TAU *n* = 61** | **BLT *n* = 29** | **EI *n* = 13** | **Total *n* = 103** |
| --- | --- | --- | --- | --- |
| Mean +/- SD  Median  P25, p75  Min, Max  Missing | -2.1 +/-9.8  -1.0  -7.0, 4.0  -25.0, 29.2  3 | 1.4 +/-7.8  3.0  -1.0, 6.4  -15.7, 14.0  0 | -2.7 +/-5.5  -3.5  -5.3, 1.0  -15.0, 7.0  0 | -1.1 +/-8.9  0.0  -5.2, 4.5  -25.0, 29.2  3 |

**Supplementary Table 17** Statistical results for the primary outcome (IDS-C_30_ total score, difference T4-T2)

|  | | **TAU** | | **BLT** | | **EI** | |  |
| --- | --- | --- | --- | --- | --- | --- | --- | --- |
|  | ***n*** | **LSM** | **95% CI** | **LSM** | **95% CI** | **LSM** | **95% CI** | ***p*-value** |
| mITT analysis | 174 | -1.428 | [-3.381,0.526] | -0.124 | [-2.219,1.971] | -2.646 | [-4.777,-0.515] | 0.2384 |
| Adherence to intervention as covariate^a^ | 105 | n/a | n/a | -0.113 | [-2.127,1.902] | -2.632 | [-4.643,-0.621] | 0.088 |
| Per protocol analysis | 57 | -1.047 | [-3.71,1.616] | 2.816 | [-2.54,8.173] | -5.802 | [-14.575,2.97] | 0.1996 |
| Additional per protocol analysis – version 1 | 76 | -0.813 | [-3.267,1.641] | 1.844 | [-1.502,5.19] | -3.548 | [-8.745,1.649] | 0.1757 |
| Additional per protocol analysis – version 2 | 103 | -1.307 | [-3.31,0.695] | 0.947 | [-1.955,3.848] | -2.644 | [-6.991,1.703] | 0.2864 |
| Complete case analysis | 159 | -1.469 | [-3.432,0.494] | -0.119 | [-2.258,2.019] | -2.613 | [-4.76,-0.466] | 0.2481 |
| LOC analysis | 174 | -1.328 | [-3.216,0.56] | -0.287 | [-2.247,1.674] | -2.574 | [-4.595,-0.553] | 0.2576 |

^a^Only participants randomised to BLT or AEI are considered in this analysis, since adherence to intervention is only measured for these groups. Adherence to intervention is included as percentage of conducted sessions in the model. The analysis of covariance revealed no effect of adherence to intervention (*p* = 0.8526).

|  | **BLT - TAU** | | **EI - TAU** | | **BLT - EI** | |
| --- | --- | --- | --- | --- | --- | --- |
|  | **LSM** | **95% CI** | **LSM** | **95% CI** | **LSM** | **95% CI** |
| mITT analysis | 1.304 | [-1.537,4.144] | -1.219 | [-4.09,1.652] | 2.522 | [-0.408,5.453] |
| Adherence to intervention as covariate | n/a | n/a | n/a | n/a | 2.52 | [-0.382,5.422] |
| Per protocol analysis | 3.863 | [-2.125,9.852] | -4.755 | [-14.168,4.657] | 8.619 | [-1.543,18.781] |
| Additional per protocol analysis – version 1 | 2.657 | [-1.396,6.71] | -2.735 | [-8.57,3.101] | 5.392 | [-0.696,11.479] |
| Additional per protocol analysis – version 2 | 2.254 | [-1.217,5.724] | -1.337 | [-6.161,3.488] | 3.591 | [-1.535,8.717] |
| Complete case analysis | 1.350 | [-1.517,4.216] | -1.144 | [-4.025,1.736] | 2.494 | [-0.448,5.436] |
| LOC analysis | 1.041 | [-1.644,3.726] | -1.246 | [-3.986,1.494] | 2.287 | [-0.445,5.019] |

**Supplementary Table 18** Statistical results for secondary outcomes based on the mITT set

|  | | **TAU** | | **BLT** | | **EI** | |  |
| --- | --- | --- | --- | --- | --- | --- | --- | --- |
|  | ***n*** | **LSM** | **95% CI** | **LSM** | **95% CI** | **LSM** | **95% CI** | ***p*-value** |
| IDS-C_30_ total score (T5-T2) | 174 | -0.411 | [-2.531,1.709] | 0.940 | [-1.256,3.136] | -2.172 | [-4.385,0.04] | 0.1351 |
| BDI-II (T4-T2) | 171 | -4.234 | [-6.221,-2.247 | -4.526 | [-6.656,-2.396] | -4.999 | [-7.172,-2.827] | 0.8724 |
| BDI-II (T5-T2) | 171 | -4.086 | [-6.222,-1.95] | -2.410 | [-4.609,-0.211] | -4.964 | [-7.198,-2.73] | 0.2500 |
| ADHD Rating Scale total score (T4-T2) | 173 | -2.834 | [-4.562,-1.105] | -2.794 | [-4.634,-0.955] | -2.886 | [-4.728,-1.045] | 0.9974 |
| ADHD Rating Scale total score (T5-T2) | 173 | -3.091 | [-5.035,-1.147] | -3.414 | [-5.406,-1.421] | -3.321 | [-5.317,-1.325] | 0.9720 |
| **BMI (T4 – T2)** | **172** | **0.409** | **[0.178,0.64]** | **-0.033** | **[-0.277,0.212]** | **0.236** | **[-0.011,0.483]** | **0.0349** |
| BMI (T5 - T2) | 172 | 0.501 | [0.165,0.837] | 0.291 | [-0.05,0.631] | 0.318 | [-0.02,0.657] | 0.6374 |
| Body fat percentage (T4-T2) | 173 | 0.462 | [-0.115,1.04] | -0.006 | [-0.634,0.621] | 0.282 | [-0.34,0.904] | 0.5470 |
| Body fat percentage (T5-T2) | 173 | 0.748 | [-0.011,1.506] | -0.037 | [-0.8,0.725] | 0.746 | [-0.007,1.499] | 0.2464 |
| Waist circumference (T4-T2) | 172 | 1.057 | [-0.188,2.303] | -0.922 | [-2.267,0.423] | -0.606 | [-1.963,0.751] | 0.0708 |
| Waist circumference (T5-T2) | 172 | 1.274 | [-0.495,3.043] | 1.618 | [-0.196,3.433] | 0.683 | [-1.132,2.498] | 0.7614 |
| Waist-to-hip ratio (T4-T2) | 172 | -0.002 | [-0.016,0.011] | -0.016 | [-0.031,-0.002] | -0.006 | [-0.021,0.009] | 0.3544 |
| Waist-to-hip ratio (T5-T2) | 172 | 0.001 | [-0.018,0.02] | 0.004 | [-0.015,0.024] | 0.004 | [-0.015,0.024] | 0.9677 |

|  | **BLT - TAU** | | **EI - TAU** | | **BLT - EI** | |
| --- | --- | --- | --- | --- | --- | --- |
|  | **LSM** | **95% CI** | **LSM** | **95% CI** | **LSM** | **95% CI** |
| IDS-C_30_ total score (T5-T2) | 1.351 | [-1.682,4.384] | -1.761 | [-4.814,1.291] | 3.113 | [0.051,6.174] |
| BDI-II (T4-T2) | -0.292 | [-3.169,2.585] | -0.765 | [-3.675,2.144] | 0.473 | [-2.491,3.438] |
| BDI-II (T5-T2) | 1.676 | [-1.36,4.712] | -0.878 | [-3.945,2.189] | 2.554 | [-0.519,5.628] |
| ADHD Rating Scale total score (T4-T2) | 0.039 | [-2.444,2.523] | -0.053 | [-2.557,2.452] | 0.092 | -2.454,2.638] |
| ADHD Rating Scale total score (T5-T2) | -0.323 | [-3.079,2.434] | -0.230 | [-3.004,2.544] | -0.092 | [-2.868,2.684] |
| **BMI (T4 – T2)** | **-0.442** | **[-0.776,-0.107]** | **-0.173** | **[-0.511,0.164]** | **-0.268** | **[-0.612,0.076]** |
| BMI (T5 - T2) | -0.211 | [-0.688,0.267] | -0.183 | [-0.659,0.293] | -0.028 | [-0.505,0.45] |
| Body fat percentage (T4-T2) | -0.468 | [-1.313,0.376] | -0.180 | [-1.025,0.664] | -0.288 | [-1.159,0.583] |
| Body fat percentage (T5-T2) | -0.785 | [-1.854,0.285] | -0.001 | [-1.067,1.064] | -0.783 | [-1.843,0.277] |
| Waist circumference (T4-T2) | -1.979 | [-3.794,-0.165] | -1.663 | [-3.5,0.174] | -0.316 | [-2.183,1.55] |
| Waist circumference (T5-T2) | 0.344 | [-2.175,2.863] | -0.591 | [-3.121,1.938] | 0.935 | [-1.595,3.466] |
| Waist-to-hip ratio (T4-T2) | -0.014 | [-0.034,0.006] | -0.004 | [-0.024,0.016] | -0.010 | [-0.031,0.01] |
| Waist-to-hip ratio (T5-T2) | 0.003 | [-0.024,0.03] | 0.003 | [-0.024,0.031] | 0.000 | [-0.028,0.027] |

## **SI9. Exploratory analyses**

##

**9.1 Exploratory analyses of intervention adherence**

The following analyses were requested by one of the reviewers, to gain additional information for future studies and the clinical application of both interventions in individuals with ADHD.

**9.1.1 Statistical analyses**

The association of baseline demographic and clinical variables with adherence to BLT or EI was evaluated by means of a multivariable linear regression model. First, a full model was run including all candidate variables (age, sex, IQ, education of parents, BMI, psychiatric comorbidity, IDS-C_30_ total score, ADHD Rating scale total score) separately for the dependent variable adherence to BLT and adherence to EI. Missing data on the candidate variables was not imputed and a complete case analysis was run. Residuals were graphically checked for normal distribution (criterion was fulfilled) and correlations between candidate variables were evaluated (no strong correlations found). Second, variable selection was performed to yield a reduced model. Stepwise selection was applied based on the Akaike information criterion (AIC) where smaller AIC values means better model fit.

Additionally, cluster analysis was performed in the whole sample (including BLT and EI groups) based on continuous baseline variables (age, IQ, education of parents, BMI, ADHD Rating scale total score) in order to identify groups of participants with similar baseline characteristics. Variables were standardized (mean zero and variance one) before applying the cluster procedure. Clustering was then performed with the proc cluster procedure of SAS using Ward’s minimum variance cluster analysis method. Based on graphical inspection, a three cluster solution was identified. The three cluster groups were compared descriptively as well as statistically by means of a one-way ANOVA with respect to outcome variables adherence to BLT, adherence to EI, and change in depressive symptoms from baseline to T4 (IDS-C_30_ total score, difference T4-T2, calculated separately for the BLT and the EI group).

These analyses are of exploratory nature, and were not pre-specified in the study protocol.

**9.1.2 Results**

**9.1.2.1 Association between baseline demographic and clinical variables and adherence to BLT**

Lower ADHD Rating scale total scores were associated with higher adherence to BLT (full model, *estimate* = -0.8375, *t* = -1.78, *p* = .082; reduced model, *estimate* = -0.9873, *t* = -2.47 *p* = .016; see Supplementary Tables 19-20). IQ was also included in the reduced model and was negatively associated with adherence to BLT, however this association was less pronounced in the exploratory analyses (full model, *estimate* = -0.6206, *t* = -1.74, *p* = .088; reduced model, *estimate* = -0.4974, t = -1.58 *p* = .120; see Supplementary Tables 19-20). None of the other baseline variables (age, sex, education of parents, BMI, psychiatric comorbidity, IDS-C_30_ total score) were selected for the reduced model and predicted adherence to BLT.

**9.1.2.2 Association between baseline demographic and clinical variables and adherence to EI**

There was a significant positive association between age and intervention adherence in the EI group. This was revealed by the results of the full and the reduced model (all *p*-values < .0001, Supplementary Tables 21-22). Thus, adherence to EI significantly increased with increasing age in our sample of individuals with ADHD. Education of parents and BMI were also selected for the reduced model, which however revealed no significant association with intervention adherence in the present sample (education of parents; *estimate* = 3.6105, *t* = 1.31, *p* = .1952; BMI, *estimate* = -0.6568, *t* = -1.31, *p* = .1960). None of the other baseline variables (sex, IQ, psychiatric comorbidity, ADHD Rating scale total score, IDS-C_30_ total score) were selected for the reduced model and predicted adherence to EI.

**9.2. Exploratory analyses of clinical subgroups**

**9.2.1 Statistical analyses**

**9.2.2 Results of Ward’s minimum variance cluster analysis**

**9.2.2.1 Descriptive analyses of the clusters**

The cluster analysis identified three groups of patients who were characterised by differences in age and several additional demographic (i.e. IQ, education of parents) and clinical variables (i.e. BMI, ADHD symptoms) (Supplementary Table 23). Specifically, one group of older participants (cluster 3, mean age of 36.65 years) could be identified, who were also characterised by a mean IQ above average and a mean BMI in the normal range. This group differed predominantly in terms of age from a second group of younger participants (cluster 1, mean age of 22.93 years) also characterised by a mean IQ above average as well as high education of parents, and a BMI in the normal range. This group differed from a second group of younger participants (cluster 2, mean age of 24.72 years) with a mean IQ in the normal range and a high mean BMI corresponding to obesity class I. Also, ADHD symptoms were most pronounced in the latter cluster.

**9.2.2.2 Between-cluster comparisons of intervention adherence and the primary efficacy endpoint**

**BLT.** Adherence to BLT did not differ between the three clinical subgroups identified by the cluster analysis (*p* = .329, Supplementary Table 24). Depressive symptoms descriptively increased from baseline to T4 for participants of cluster 3 (mean difference of 5.1), while depressive symptoms slightly decreased for participants of the two other clusters (cluster 1, mean difference of -1.3; cluster 2, mean difference of -0.2). These changes in IDS-C_30_ total scores from baseline to T4 did not significantly differ between the clinical subgroups (*p* = .145, Supplementary Table 24).

**EI.** There was a significant group difference for adherence to EI (*p* = .002) indicating higher intervention adherence (mean adherence, 51.2%) of participants from cluster 3 characterised by higher age compared to both groups of younger participants (cluster 1, mean adherence, 23.1%; cluster 2, mean adherence, 19.1%, Supplementary Table 24). Although participants from cluster 3 showed higher intervention adherence, reductions in IDS-C_30_ total scores from baseline to T4 (cluster 1, mean difference of -3.7; cluster 2, mean difference of -1.0, cluster 3, mean difference of -1.4) were comparable across the clinical subgroups (*p* = .398, Supplementary Table 24).

**Supplementary Table 19** Linear regression model testing the association between demographic and clinical variables at baseline and adherence to BLT: Results of the full model

| **Model fit** | **AIC**  533.8 |  |  |  |  |
| --- | --- | --- | --- | --- | --- |
| **Solution for fixed effects** | | | | | |
| **Effect** | ***Estimate*** | ***SE*** | ***F*** | ***t*** | ***Pr > \|t\|*** |
| Intercept | 124.97 | 48.75 | 49 | 2.56 | 0.0135 |
| Age | 0.0403 | 0.05 | 49 | 0.82 | 0.4163 |
| Sex (female) | 7.3492 | 8.82 | 49 | 0.83 | 0.4090 |
| IQ | -0.6206 | 0.36 | 49 | -1.74 | 0.0878 |
| Education of parents | 2.2904 | 4.61 | 49 | 0.50 | 0.6217 |
| BMI | -0.6619 | 0.72 | 49 | -0.92 | 0.3616 |
| At least one comorbid psychiatric disorder (no) | 10.6386 | 10.88 | 49 | 0.98 | 0.3331 |
| ADHD Rating scale total score | -0.8375 | 0.47 | 49 | -1.78 | 0.0821 |
| IDS-C_30_ total score | -0.0244 | 0.54 | 49 | -0.05 | 0.9639 |

The linear regression model (number of observations used = 58) included adherence to BLT as dependent variable and age, sex (female, male), IQ, highest education of parents, BMI, at least on comorbid psychiatric condition (yes/ no), ADHD Rating scale total score, and IDS-C_30_ total score as predictors. Adherence to BLT was defined as the percentage of conducted BLT sessions as indicated by self-reports recorded online with the m-health app. Parental education status represents the mean of the biological father’s and mother’s ISCED score. AIC = Akaike information criterion, BMI = body mass index, IDS-C_30_ = Inventory of Depressive Symptomatology, ISCED = International Standard Classification of Education.

**Supplementary Table 20** Linear regression model testing the association between demographic and clinical variables at baseline and adherence to BLT: Results of the model with a reduced number of variables

| **Model fit** | **AIC**  569.2 |  |  |  |  |
| --- | --- | --- | --- | --- | --- |
| **Solution for fixed effects** | | | | | |
| **Effect** | ***Estimate*** | ***SE*** | ***DF*** | ***t*** | ***Pr > \|t\|*** |
| Intercept | 133.47 | 36.72 | 57 | 3.63 | 0.0006 |
| IQ | -0.4974 | 0.32 | 57 | -1.58 | 0.1202 |
| **ADHD Rating scale total score** | **-0.9873** | **0.40** | **57** | **-2.47** | **0.0164** |

The linear regression model (number of observations used = 60) included adherence to BLT as dependent variable and IQ and ADHD Rating scale total score as predictors. Predictors were selected using the Akaike information criterion (AIC). Adherence to BLT was defined as the percentage of conducted BLT sessions as indicated by self-reports recorded online with the m-health app.

**Supplementary Table 21** Linear regression model testing the association between demographic and clinical variables at baseline and adherence to EI: Results of the full model

| **Model fit** | **AIC**  486.0 |  |  |  |  |
| --- | --- | --- | --- | --- | --- |
| **Solution for fixed effects** | | | | | |
| **Effect** | ***Estimate*** | ***SE*** | ***DF*** | ***t*** | ***Pr > \|t\|*** |
| Intercept | 2.2822 | 33.18 | 46 | 0.07 | 0.9455 |
| **Age** | **0.1587** | **0.04** | **46** | **4.35** | **<0.0001** |
| Sex (female) | 0.5976 | 7.50 | 46 | 0.08 | 0.9368 |
| IQ | -0.3381 | 0.35 | 46 | -0.96 | 0.3412 |
| Education of parents | 5.6334 | 3.53 | 46 | 1.60 | 0.1171 |
| BMI | -0.6830 | 0.53 | 46 | -1.28 | 0.2057 |
| At least one comorbid psychiatric disorder (no) | 3.0565 | 8.06 | 46 | 0.38 | 0.7063 |
| ADHD Rating scale total score | 0.1464 | 0.49 | 46 | 0.30 | 0.7651 |
| IDS-C30 total score | 0.2347 | 0.44 | 46 | 0.53 | 0.5992 |

| **Model fit** | **AIC**  536.2 |  |  |  |  |
| --- | --- | --- | --- | --- | --- |
| **Solution for fixed effects** | | | | | |
| **Effect** | ***Estimate*** | ***SD*** | ***DF*** | ***t*** | ***Pr > \|t\|*** |
| Intercept | -16.44 | 20.96 | 55 | -0.78 | 0.4362 |
| **Age** | **0.1486** | **0.03** | **55** | **5.04** | **<0.0001** |
| Education of parents | 3.6105 | 2.75 | 55 | 1.31 | 0.1952 |
| BMI | -0.6568 | 0.50 | 55 | -1.31 | 0.1960 |

The linear regression model (number of observations used = 55) included adherence to EI as dependent variable and age, sex (female, male), IQ, highest education of parents, BMI, at least on comorbid psychiatric condition (yes/ no), ADHD Rating scale total score, and IDS-C_30_ total score as predictors. Adherence to EI was defined as the percentage of conducted EI sessions as indicated by self-reports recorded online with the m-health app. Parental education status represents the mean of the biological father’s and mother’s ISCED score. AIC = Akaike information criterion, BMI = body mass index, IDS-C_30_ = Inventory of Depressive Symptomatology, ISCED = International Standard Classification of Education.

**Supplementary Table 22** Linear regression model testing the association between demographic and clinical variables at baseline and adherence to EI: Results of the model with a reduced number of variables

The linear regression model (number of observations used = 59) included adherence to EI as dependent variable and age, education of partents, and BMI as predictors. Predictors were selected using the Akaike information criterion (AIC). Adherence to EI was defined as the percentage of conducted BLT sessions as indicated by self-reports recorded online with the m-health app. Parental education status represents the mean of the biological father’s and mother’s ISCED score. BMI = body mass index, ISCED = International Standard Classification of Education.

**Supplementary Table 23** Results of Ward’s minimum variance cluster analysis: Descriptive results

| **Variable** | **Cluster 1**  ***n = 53*** | **Cluster 2**  ***n = 46*** | **Cluster 3**  ***n = 32*** |
| --- | --- | --- | --- |
| **Age** (months) |  |  |  |
| Mean +/- SD | 275.17 +/- 53.41 | 296.63 +/- 88.84 | **439.75 +/- 76.00** |
| Min, Max | 174.00, 375.00 | 173.00, 520.00 | 295.00, 539.00 |
| **IQ** |  |  |  |
| Mean +/- SD | 112.48 +/- 9.34 | **98.68 +/- 10.38** | 113.67 +/- 12.16 |
| Min, Max | 85.00, 129.25 | 75.00, 123.25 | 92.50, 140.00 |
| **Education of parents** |  |  |  |
| Mean +/- SD | 4.74 +/- 0.81 | 3.39 +/- 0.83 | 3.05 +/- 1.22 |
| Min, Max | 3.00, 6.00 | 1.50, 5.00 | 1.00, 5.00 |
| **BMI** |  |  |  |
| Mean +/- SD | 22.47 +/- 3.57 | **30.15 +/- 7.16** | 24.75 +/- 3.91 |
| Min, Max | 15.91, 34.81 | 18.88, 51.12 | 18.66, 32.32 |
| **ADHD Rating scale total score** |  |  |  |
| Mean +/- SD | 25.92 +/-7.78 | **27.91 +/- 10.74** | 24.22 +/- 7.65 |
| Min, Max | 10.00, 43.00 | 8.00, 45.00 | 6.00, 38.00 |
| **IDS-C_30_ total score^1^** |  |  |  |
| Mean +/- SD | 11.34 +/- 7.19 | 12.73 +/- 7.82 | **14.11 +/- 10.41** |
| Min, Max | 1.00, 36.00 | 4.00, 40.00 | 0.00, 38.00 |

Clustering was based on age (months), IQ, education of parents, BMI, and ADHD Rating scale total score. ^1^Cluster analysis did not include IDS-C_30_ total score. Parental education status represents the mean of the biological father’s and mother’s ISCED score. BMI = body mass index, IDS-C_30_ = Inventory of Depressive Symptomatology, ISCED = International Standard Classification of Education.

| **Variable** | **Cluster 1** | | | **Cluster 2** | | **Cluster 3** | | | ***p-value*** |
| --- | --- | --- | --- | --- | --- | --- | --- | --- | --- |
|  | | |  | |  | |  |  |  |
| **Adherence to BLT** | | |  | |  | |  |  |  |
| *n* | | 24 | | 24 | | 13 | | | 0.329 |
| Mean +/- SD | | 56.5 +/- 28.2 | | 43.9 +/- 30.7 | | 55.5 +/- 37.0 | | |  |
| Median | | 52.5 | | 40.8 | | 61.7 | | |  |
| p25, p75 | | 41.7, 75.8 | | 12.5, 70.0 | | 23.3, 85.0 | | |  |
| Min, Max | | 6.7, 105.0 | | 1.7, 98.3 | | 1.7, 113.3 | | |  |
| **Adherence to EI** | |  | |  | |  | | |  |
| *n* | | 24 | | 18 | | 14 | | | **0.002** |
| Mean +/- SD | | 23.1 +/-26.8 | | 19.1 +/- 17.9 | | 51.2 +/- 31.8 | | |  |
| Median | | 12.5 | | 12.5 | | 47.5 | | |  |
| p25, p75 | | 2.5, 30.8 | | 7.5, 27.5 | | 31.7, 67.5 | | |  |
| Min, Max | | 0, 100.0 | | 0, 61.7 | | 1.7, 115.0 | | |  |
| **IDS-C_30_ total score, difference T4-T2, BLT** | |  | |  | |  | | |  |
| *n* | | 22 | | 18 | | 11 | | | 0.145 |
| Mean +/- SD | | -1.3 +/- 7.1 | | -0.2 +/- 10.6 | | 5.1 +/- 8.8 | | |  |
| Median | | 0 | | 0.9 | | 7.0 | | |  |
| p25, p75 | | -5.0, 3.0 | | -11.8, 6.4 | | -2.1, 11.9 | | |  |
| Min, Max | | -21.0, 8.6 | | -17.0, 18.3 | | -8.0, 20.0 | | |  |
| **IDS-C_30_ total score, difference T4-T2, EI** | |  | |  | |  | | |  |
| *n* | | 20 | | 12 | | 15 | | | 0.398 |
| Mean +/- SD | | -3.7 +/- 7.6 | | -1.0 +/- 5.1 | | -1.4 +/- 4.6 | | |  |
| Median | | -2.5 | | -3.0 | | -1.0 | | |  |
| p25, p75 | | -7.0, 1.5 | | -4.3, 3.3 | | -4.0, 2.0 | | |  |
| Min, Max | | -22.3, 8.0 | | -7.0, 8.0 | | -11.0, 7.0 | | |  |

**Supplementary Table 24** Results of Ward’s minimum variance cluster analysis: Between-cluster comparisons of outcome measures (adherence to BLT, adherence to EI, change in depressive symptoms from baseline to T4)

## **SI9. References**

1. Mayer JS, Hees K, Medda J, Grimm O, Asherson P, Bellina M, Colla M, Ibáñez P, Koch E, Martinez-Nicolas A, Muntaner-Mas A, Rommel A, Rommelse N, Ruiter S de, Ebner-Priemer UW, Kieser M, Ortega FB, Thome J, Buitelaar JK, Kuntsi J, Ramos-Quiroga JA, Reif A, Freitag CM (2018) Bright light therapy versus physical exercise to prevent co-morbid depression and obesity in adolescents and young adults with attention-deficit / hyperactivity disorder: study protocol for a randomized controlled trial. Trials 19(1):140. https://doi.org/10.1186/s13063-017-2426-1

2. Jordan PW, Thomas B, Weerdmeester BA, McClelland AL (1996) Usability Evaluation in Industry. Taylor and Francis, London

3. Kaufman J, Birmaher B, Brent D, Rao U, Flynn C, Moreci P, Williamson D, Ryan N (1997) Schedule for Affective Disorders and Schizophrenia for School-Age Children-Present and Lifetime Version (K-SADS-PL): initial reliability and validity data. Journal of the American Academy of Child and Adolescent Psychiatry 36(7):980–988. https://doi.org/10.1097/00004583-199707000-00021

4. Kooij JJS (2010) Adult ADHD. Diagnostic assessment and treatment. Pearson Assessment and Information BV, Amsterdam

5. Kessler RC, Adler L, Ames M, Demler O, Faraone S, Hiripi E, Howes MJ, Jin R, Secnik K, Spencer T, Ustun TB, Walters EE (2005) The World Health Organization Adult ADHD Self-Report Scale (ASRS): a short screening scale for use in the general population. Psychological Medicine 35(2):245–256. https://doi.org/10.1017/s0033291704002892

6. Marchant BK, Reimherr FW, Robison D, Robison RJ, Wender PH (2013) Psychometric properties of the Wender-Reimherr Adult Attention Deficit Disorder Scale. Psychological Assessment 25(3):942–950. https://doi.org/10.1037/a0032797

7. Wittchen HU, Zaudig M, Fydrich T (1997) SKID Strukturiertes Klinisches Interview für DSM-IV Achse I und II Handanweisung. Hogrefe, Göttingen

8. Wechsler D (2008) Wechsler Adult Intelligence Scale - Fourth Edition (WAIS). Pearson Assessment, Frankfurt am Main

9. Wechsler D (2003) Wechsler Intelligence Scale for Children - Fourth Edition (WISC). Pearson Assessment, Frankfurt am Main

10. Sykes K, Roberts A (2004) The Chester step test—a simple yet effective tool for the prediction of aerobic capacity. Physiotherapy 90(4):183–188. https://doi.org/10.1016/j.physio.2004.03.008

11. Horne JA, Ostberg O (1976) A self-assessment questionnaire to determine morningness-eveningness in human circadian rhythms. International Journal of Chronobiology 4(2):97–110

12. Shrout PE, Fleiss JL (1979) Intraclass correlations: uses in assessing rater reliability. Psychological Bulletin 86(2):420–428. https://doi.org/10.1037//0033-2909.86.2.420

13. R Core Team (2019) R: A Language and Environment for Statistical Computing. R Foundation for Statistical Computing, Vienna

14. Rush AJ, Gullion CM, Basco MR, Jarrett RB, Trivedi MH (1996) The Inventory of Depressive Symptomatology (IDS): psychometric properties. Psychological Medicine 26(3):477–486. https://doi.org/10.1017/s0033291700035558

15. Drieling T, Schärer LO, Langosch JM (2007) The Inventory of Depressive Symptomatology: German translation and psychometric validation. International Journal of Methods in Psychiatric Research 16(4):230–236. https://doi.org/10.1002/mpr.226

16. Helmreich I, Wagner S, Mergl R, Allgaier A-K, Hautzinger M, Henkel V, Hegerl U, Tadic A (2011) The Inventory Of Depressive Symptomatology (IDS-C(28)) is more sensitive to changes in depressive symptomatology than the Hamilton Depression Rating Scale (HAMD(17)) in patients with mild major, minor or subsyndromal depression. European Archives of Psychiatry and Clinical Neuroscience 261(5):357–367

17. Beck AT, Steer RA, Brown GK (1996) Manual for the Beck depression inventory-II. Psychological Corporation, San Antonio, TX

18. Dupaul GJ (1998) ADHD rating scale-IV: Checklists, norms, and clinical interpretation. The Guilford Press, New York

19. Barkley RA (2011) Barkley Adult ADHD Rating Scale-IV (BAARS-IV). The Guilford Press, New York

20. Brozek J, Grande F, Anderson JT, Keys A (1963) Densitometric analysis of body composition: Revision of some quantitative assumptions. Annals of the New York Academy of Sciences 110:113–140
